# The impact of sequencing depth and relatedness of the reference genome in population genomic studies: A case study with two caddisfly species (Trichoptera, Rhyacophilidae, *Himalopsyche*)

**DOI:** 10.1002/ece3.9583

**Published:** 2022-12-12

**Authors:** Xi‐Ling Deng, Paul B. Frandsen, Rebecca B. Dikow, Adrien Favre, Deep Narayan Shah, Ram Devi Tachamo Shah, Julio V. Schneider, Jacqueline Heckenhauer, Steffen U. Pauls

**Affiliations:** ^1^ Senckenberg Research Institute and Natural History Museum Frankfurt/Main Germany; ^2^ Institute of Insect Biotechnology Justus‐Liebig‐University Gießen Gießen Germany; ^3^ LOEWE Centre for Translational Biodiversity Genomics (LOEWE‐TBG) Frankfurt/Main Germany; ^4^ Department of Plant & Wildlife Sciences Brigham Young University Provo Utah USA; ^5^ Data Science Lab, Office of the Chief Information Officer Smithsonian Institution Washington DC USA; ^6^ Regional Nature Park of the Trient Valley Salvan Switzerland; ^7^ Central Department of Environmental Science Tribhuvan University Kirtipur Nepal; ^8^ Aquatic Ecology Centre, School of Science Kathmandu University Dhulikhel Nepal; ^9^ Department of Life Sciences School of Science, Kathmandu University Dhulikhel Nepal

**Keywords:** aquatic insects, de novo genomes, population genomics, reference genomes, sequencing depth, whole genome resequencing

## Abstract

Whole genome sequencing for generating SNP data is increasingly used in population genetic studies. However, obtaining genomes for massive numbers of samples is still not within the budgets of many researchers. It is thus imperative to select an appropriate reference genome and sequencing depth to ensure the accuracy of the results for a specific research question, while balancing cost and feasibility. To evaluate the effect of the choice of the reference genome and sequencing depth on downstream analyses, we used five confamilial reference genomes of variable relatedness and three levels of sequencing depth (3.5×, 7.5× and 12×) in a population genomic study on two caddisfly species: *Himalopsyche digitata* and *H. tibetana*. Using these 30 datasets (five reference genomes × three depths × two target species), we estimated population genetic indices (inbreeding coefficient, nucleotide diversity, pairwise *F*_ST_, and genome‐wide distribution of *F*_ST_) based on variants and population structure (PCA and admixture) based on genotype likelihood estimates. The results showed that both distantly related reference genomes and lower sequencing depth lead to degradation of resolution. In addition, choosing a more closely related reference genome may significantly remedy the defects caused by low depth. Therefore, we conclude that population genetic studies would benefit from closely related reference genomes, especially as the costs of obtaining a high‐quality reference genome continue to decrease. However, to determine a cost‐efficient strategy for a specific population genomic study, a trade‐off between reference genome relatedness and sequencing depth can be considered.

## Graphical abstract

To investigate the impacts of the reference genome and sequencing depth in population genomic studies, we compared results of population genetic indices and population structure derived from three levels of sequencing depth and five confamilial reference genomes of variable relatedness. Our study demonstrated that both distantly related reference genomes and lower sequencing depth lead to less accurate results. However, choosing a more closely related reference genome may significantly remedy the defects caused by low sequencing depth, which indicates that population genomic studies would benefit most from closely related reference genomes.

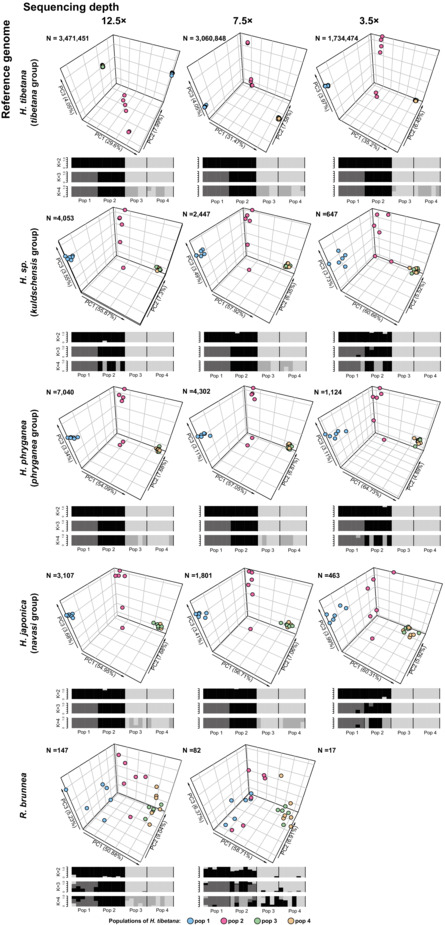

## 1 INTRODUCTION

As high‐throughput sequencing (HTS) technologies and bioinformatic tools are rapidly becoming more accurate and increasingly affordable, it is possible to generate whole genome resequencing (WGR) data for almost any species. High‐quality WGR data provide a remarkable amount of information, including a vast number of loci as well as a large number of genetic variants, thus enabling powerful population genomics analyses (Goodwin et al., 2016). Today, whole genome resequencing with low read depth, which indicates a low average number of reads that are aligned to a base in the reference genome, is widely applied in population studies (Lou et al., 2021; Nielsen et al., 2011; Sims et al., 2014). When initiating a project on population genetics using WGR, there are two prerequisites: (i) the availability of a reference genome representing the focal species (Ellegren, 2014) and (ii) estimating the necessary sequencing depth to support accurate results (Meisner & Albrechtsen, 2018).

The selection of reference genome and sequencing depth are therefore two important features in a population genetic study. However, with a fixed budget, it is important to find a balance between data quality and sequencing costs by compromising on the reference genome or sequencing depth. For every study, the reference genome needs to be carefully chosen to avoid bias in mapping and variant calling, considering the amount of sequence identity between the reference genome and the data resulting from resequencing (Nielsen et al., 2011). Ideally, such studies should include a high‐quality species‐specific reference genome, which is often not available for nonmodel organisms. Given the costs and time associated with generating a de novo reference genome, it can be more realistic to use an existing one, yet more distantly related, as is done most often in population genomic studies (Duchen & Salamin, 2021). Currently, several empirical studies have examined the impact of nonconspecific reference genomes in population genomics. For example, Gopalakrishnan et al. (2017) compared the use of dog and wolf genomes as reference genomes for either domestic dog or wolf populations. Their results showed that the selection of the reference genome only had a minor influence on downstream analyses, probably because of the close relatedness of these two taxa, which diverged approx. 30,000 years ago (Skoglund et al., 2015; Wang et al., 2013). By contrast, other studies have found that the use of distantly related reference genomes biases the results of resequencing analyses in bacteria (Valiente‐Mullor et al., 2021), fungi (Garcia‐Rubio et al., 2018), and mammals (Yang et al., 2019), but no studies are yet available for insects.

In comparison to choosing the reference genome based upon relatedness, sequencing depth needs more preliminary knowledge to determine. The sequencing depth needs to be tailored to each particular study based on many aspects, for instance genome size of the target species, the availability of funding, and of course the research question. Using the strategy of low sequencing depth in a population genetic study may result in data loss, thus causing statistical uncertainty during genotype and variant calling (Crawford & Lazzaro, 2012; Meisner & Albrechtsen, 2018; Nielsen et al., 2012). This statistical uncertainty is mainly due to the limited information provided by the low amount of reads, leading to poor discrimination between sequencing error and real variation, that is, SNPs (Meisner & Albrechtsen, 2018). To improve the accuracy of estimates in a cost‐limited population genetic study, especially using low sequencing depth, some researchers therefore demonstrated that employing a large sample size provides more accurate results, for instance, more than 50 individuals from a population with a sequencing depth of two (Buerkle & Gompert, 2013; Fumagalli, 2013; Han et al., 2014; Sims et al., 2014). However, unlike these studies that are based on simulated data, it is challenging to obtain a large number of samples for taxa such as the aquatic insect genus *Himalopsyche*. These species are apex predators in the benthic invertebrate community and naturally have small population sizes. Many other taxa are similarly rare. Thus, it is pragmatic to consider how we might improve population‐based inference through the optimization of reference genome choice or sequencing depth for these sample‐limited studies. Therefore, comprehensively exploring the influence of reference choice and sequencing depth on downstream analyses is urgently needed, particularly for insects. In this study, we will evaluate the effects of the reference genome and sequencing depth on a genus of caddisflies, for which we already have significant molecular and taxonomic data (Deng et al., 2021; Heckenhauer et al., 2022).

*Himalopsyche* is a genus of caddisflies (Insecta: Trichoptera) that is primarily distributed in the mountainous areas of central and east Asia (Hjalmarsson et al., 2018). Their larvae live as free‐roaming predators in cool fast‐flowing rivers and streams and are regarded as bioindicators of water quality (Hjalmarsson, 2019; Morse et al., 2019; Tsuruishi et al., 2006). There are currently 56 named species of *Himalopsyche* (Hjalmarsson, 2019), which are divided into five species groups: the *tibetana* group, the *lepcha* group, the *kuldschensis* group, the *phryganea* group, and the *navasi* group (Hjalmarsson et al., 2019). The species *H. digitata* and *H. tibetana* both belong to the *tibetana* group, which is distributed in the Himalayas. This area is characterized by a number of parallel north–south running river systems (such as the Karnali, the Narayani, and the Koshi) with sharp elevational gradients. Currently, climate change is causing a number of cascading effects on river flow via rapidly receding glaciers greatly affecting aquatic biodiversity (Xu et al., 2009). It is therefore crucial to investigate the patterns of genetic diversity of aquatic insects in the region to understand the current and past ecological processes, in order to promote the conservation of freshwater biodiversity (Geist, 2011). Genome‐wide analysis is an important conservation tool that can provide novel insights essential for identifying, for example, hotspots or reservoirs of genetic diversity, dispersal routes, ecological corridors, and stepping stone habitats (Barbosa et al., 2018; Brandies et al., 2019; Hohenlohe et al., 2021; Jasper et al., 2019). However, the quality and quantity of aquatic insect genomes are still relatively low compared with terrestrial insects (Hotaling et al., 2020, 2021). Despite Trichoptera covering ~275 million years of evolution (Thomas et al., 2020) and comprising ~16,300 known species (Morse et al., 2019), only 29 Trichoptera genome assemblies (26 species) have been published to date (Heckenhauer et al., 2019, 2022; Luo et al., 2018; Olsen et al., 2021; Ríos‐Touma et al., 2022). This represents <0.15% of all known species, which limits progress in genomics‐based research of this ecologically relevant group.

The number of studies using population genomics is rapidly increasing. With it, the need to test the effect of reference genome's selection and sequencing depth on the results. Indeed, such studies will allow to find a balance between data quality and sequencing costs by compromising on the reference genome or sequencing depth. Therefore, we conducted a case study using an empirical dataset to evaluate how reference genome selection, that is, degree of relatedness, and sequencing depth affect downstream population genetic analyses of the species *H. digitata* and *H. tibetana*. In addition, we tried to reveal the correlation between the inferences from population genetics and drainage network and provided insights for local biodiversity conservation of these enigmatic species.

## 2 MATERIALS AND METHODS

### 2.1 Study design

**FIGURE 1:**
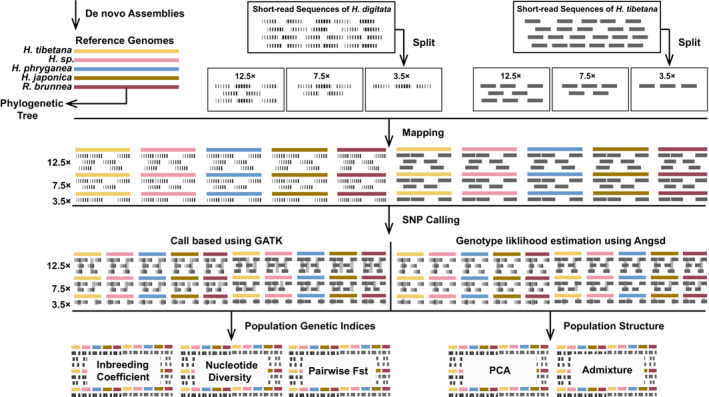
Workflow of data processing in this study showing the treatment of short‐read resequencing data from the two target species and subsequent mapping and variant calling with different reference genomes for assessing genetic diversity and population genetic structure (see details in Section 2.1).

As illustrated in Figure 1, first, we generated three new de novo whole genome assemblies for *H. tibetana* (*tibetana* group), *H. japonica* (*navasi* group), and *H*. sp. (*kuldschensis* group) sensu Hjalmarsson et al., 2019, in addition to two previously published genomes (i.e., *H. phryganea* (*phryganea* group) and *R. brunnea*; Heckenhauer et al., 2022). Since the specimens of *Himalopsyche sp.* was collected as a larva that cannot be identified to species level by morphologic diagnosis, and the CO1 sequence of this specimen differed slightly from all hitherto known sequences of named species, we can only classify this specimen as a species belonging to the *kuldschensis* group. These five genomes represent a gradient of genetic relatedness with respect to our target species, which were used as reference genomes. We used populations of two *Himalopsyche* species (*H. digitata* and *H. tibetana*), each species including four populations with each population containing six individuals except for one population from *H. digitata* and *H. tibetana*, respectively, which contained only five individuals. The reads of the populations were subsampled into three separate datasets with an average depth of 12.5×, 7.5× and 3.5×, respectively. Reads from each dataset were mapped to the five different reference genomes separately. Afterward, variants were called from all datasets using two strategies: Genotype calling with GATK and direct estimation of the genotype likelihood using ANGSD. Variants identified with the first strategy were used to calculate the population genetic indices including inbreeding coefficient (*F*), nucleotide diversity (π), and pairwise fixation index (*F*_ST_); variants estimated from the second strategy were used in the principal component analysis (PCA) and admixture analysis. Finally, we compared population genetic indices and population structure with different references and sequencing depths.

### 2.2 De novo genomes of three reference species

We used the genome assemblies of four species of *Himalopsyche* (*H. tibetana*, *H*. sp., *H. phryganea*, and *H. japonica*) and of one species from the closely related genus *Rhyacophila* (*R. brunneae*, both family Rhyacophilidae) as reference genomes. The *Himalopsyche* species represent the four major taxonomic groups in the genus according to Hjalmarsson et al. (2018): the *tibetana* group (*H. tibetana*), the *kuldschensis* group (*H*. sp.), the *phryganea* group (*H. phryganea*), and the *navasi* group (*H. japonica*). Unfortunately, we could not obtain a sample of *H. lepcha* (the only species in the *lepcha* group). *Rhyacophila* is the most closely related genus to *Himalopsyche* (Thomas et al., 2020). The genomes of *H. phryganea* (JAGVSL000000000) and *Rhyacophila brunnea* (previous version of JAGYXB000000000, available at https://doi.org/10.6084/m9.figshare.c.6033011.v1) were previously sequenced and assembled (Heckenhauer et al., 2022). We generated new de novo assemblies of *H. tibetana* (collected from the Ê Ghunsa River, Nepal), *H*. sp. (*kuldschensis* group, collected from the Ê Ghunsa River, Nepal), and *H. japonica* (collected from the Nogami River, Kiso‐machi, Nagano Prefecture, Japan). Tissue of abdomen and thorax segments were used for DNA extraction after removal of the intestinal tract. We extracted high molecular weight genomic DNA using a salting‐out protocol adapted from Miller et al. (1988), as described in Heckenhauer et al. (2019). We quantified the DNA using a Qubit 4.0 fluorometer with the dsDNA Broad Range Kit (ThermoFisher Scientific) and checked its purity with a DS11 spectrophotometer (DeNovix). We used a low‐cost sequencing strategy that has been shown to produce contiguous genome assemblies, that is, employing a combination of short (Illumina) and long‐read (Oxford Nanopore) technologies to sequence the three new reference genomes, as described in Appendix S1 (Section 1.1).

We conducted a long‐read assembly of the Oxford Nanopore Technology sequencing reads with wtdbg2 v2.4 (Ruan & Li, 2020), followed by mapping and polishing with long reads with Minimap2 v14 (Li, 2018) and Racon v1.3.1 (Vaser et al., 2017). We then performed another round of long‐read polishing with nanopolish 0.11.1 (Loman et al., 2015) by first indexing the signal‐level data in the FAST5 files using nanopolish index, realigning the long reads to the Racon‐polished assembly with minimap2, and then sorting and indexing the bam file with samtools. We used nanopolish_makerange.py to split our draft genome assembly into 50‐kb segments and generated a consensus for each segment in parallel with nanopolish variants (−‐consensus ‐‐min‐candidate‐frequency 0.1). We generated the polished genome in FASTA format using nanopolish vcf2fasta. Because noisy long reads can suffer from indel errors, even with polishing, we further polished the assembly with high‐quality short‐read data with Pilon v1.22 (Walker et al., 2014). To do this, we mapped quality trimmed Illumina reads to the “nanopolished” assembly with bwa‐mem and sorted the read alignments by leftmost coordinates using the sort options in SAMtools v1.9 (Li et al., 2009). Finally, we used Pilon v1.22 (option ‐‐fix indels) to polish the assembly. Following polishing, we used purge_dups 1.2.3 (Roach et al., 2018) to purge haplotigs and overlaps in the assembly based on read depth.

For *H. japonica*, this pipeline did not meet the expected quality regarding contiguity and BUSCO completeness. Thus, we conducted a de novo hybrid assembly with the raw Illumina data together with the long reads using MaSuRCA v.3.1.1 (Zimin et al., 2013, 2017). In the config file, we specified the insert size and a standard deviation (10% of insert size) for the Illumina reads, as well as jellyfish hash size (estimated_genome_size*~long‐read coverage (equal to depth in this scenario)). All other parameters were left as defaults. We used purge_dups 1.2.3 to purge haplotigs and overlaps in the assembly based on read depth.

We calculated assembly statistics with QUAST v5.0.2 (Gurevich et al., 2013) and examined completeness with BUSCO v4.1.4 (Simão et al., 2015; Waterhouse et al., 2018) using the Endopterygota odb10 dataset with the options ‐‐long, −m = genome and ‐sp = fly. A summary of the assembly statistics and BUSCO completeness is given in Table 1. The final genome assemblies were screened for potential contaminations with taxon‐annotated GC‐coverage plots (TAGC plots) using BlobTools v1.0 (Laetsch & Blaxter, 2017). For this purpose, all preprocessed Illumina reads of the respective species were mapped against the final genome assemblies using BWA‐MEM v0.7.17‐r1188 (Li, 2013) and taxonomic assignment for BlobTools was done with blastn using ‐task megablast and ‐e‐value 1 e‐25. Details are given in Appendix S1 (Section 1.2).

**TABLE 1:** Assembly statistics of reference genomes used in this study.

Species	Accession number	Sequencing platform (depth)^d^	Assembly length bp	N50 (bp)	No of contigs	N's per 100 kb	BUSCOS %^c^	Number of proteins
*Himalopsyche japonica* ^a^	JAHFWJ000000000	Nanopore+Illumina (18× +170×)	546,840,812	2,150,202	847	0	C:97.2% [S:96.6%, D:0.6%], F:0.8%, M:2.0%, n:2124	9983
*Himalopsyche* sp.^a^	JAHFWI000000000	Nanopore+Illumina (26× +200×)	592,402,457	7,599,818	528	0	C:96.7% [S:96.3%, D:0.4%], F:1.0%, M:2.3%, n:2124	10,049
*Himalopsyche phryganea*	JAGVSL000000000	Nanopore+Illumina (36.8× +170×)	633,785,554	4,634,010	710	0	C:97.0% [S:96.5%, D:0.5%], F:1.0%, M:2.0%, n:2124	10,994
*Himalopsyche tibetana* ^a^	JAHFWH000000000	Nanopore+Illumina (24× +150×)	665,312,086	949,059	1853	0	C:96.4% [S:95.6%, D:0.8%], F:1.0%, M:2.6%, n:2124	10,994
*Rhyacophila brunne*a	JAGYXB000000000^b^	Nanopore+Illumina (19× +154×)	1,086,872,538	1,030,560	2125	0.36	C:96.0% [S:93.3%, D:2.7%], F:1.1%, M:2.9%, n:2124	10,846

^a^ This study.

^b^ In this study, we used a previous version of this assembly for SNP calling.

^c^ Based on the Endopterygota odb10 dataset (2124 genes), C: complete, S: single, D: duplicated, F: fragmented, M: missing.

^d^ Based on Genomescope2 genome size estimation.

Genome size estimation and profiling was conducted from the short‐read sequence data with GenomeScope 2.0 (Ranallo‐Benavidez et al., 2020; Vurture et al., 2017) as described in Appendix S1 (Section 1.3).

### 2.3 Annotation

We identified and classified repetitive elements de novo and generated a library of consensus sequences for each genome using RepeatModeler 2.0 (Flynn et al., 2020). We then annotated and masked repeats in each assembly with RepeatMasker 4.1.0 (http://www.repeatmasker.org) using the custom repeat library for the species generated in the previous step. After masking repeats, genes were predicted using the homology‐based gene prediction tool GeMoMa v1.6.4 (Keilwagen et al., 2016, 2019) and the two previously published species (*H. phryganea* = RG 1 and *R. brunnea* = RG 2) as reference organisms as follows: GeMoMa ‐Xmx50G GeMoMaPipeline threads=$SLURM_NPROCS outdir=<out_dir > GeMoMa.Score=ReAlign AnnotationFinalizer.r=NO o=true t=<genome to be annotated> s=own i=<name of RG 1>a=<RG 1.gff>g=<RG 1 assembly.fasta>s=own i=i=<name of RG 2>a=a=<RG 2.gff>g=g=<RG 2 assembly.fasta>.

For functional annotation of predicted genes, we first split each amino acid FASTA file into multiple files with 50 sequences each using awk and then used blastp to search against the ncbi‐blast2.9.0+ nr database with an e‐value cutoff of 10–4, −max_target_seqs set to 10 and ‐out format 6. The resulting xml files were merged using cat and then functionally annotated using the command‐line version of Blast2GO (Götz et al., 2008).

### 2.4 Species tree reconstruction

To determine the phylogenetic relatedness among the five species, we estimated a species tree using single‐copy orthologs resulting from the BUSCO analyses of the five genome assemblies with an additional species, *Glossosoma conforme*, as an outgroup (Heckenhauer et al., 2022). For each single‐copy ortholog, we generated an unaligned FASTA file with sequences from each species. We then aligned each ortholog with the MAFFT L‐INS‐i algorithm (Katoh & Standley, 2013). We selected the best‐fit substitution model for each alignment using ModelFinder (option ‐m mfp, (Kalyaanamoorthy et al., 2017)) in IQtree v.2.0.6 (Minh et al., 2020) and estimated a maximum‐likelihood tree with 1000 ultrafast bootstrap replicates (Hoang et al., 2018) with the BNNI correction (options ‐bb 1000 ‐bnni). We then generated a multispecies coalescent species tree in ASTRAL‐III (Zhang et al., 2018) using the best maximum‐likelihood tree from each ortholog as input. We visualized the trees using FigTree v.1.4.4 (http://tree.bio.ed.ac.uk/software/figtree/).

### 2.5 Cactus alignment and Hal

To further characterize the variation among the five genomes, we computed a whole genome alignment using Cactus v1.0.0 (Armstrong et al., 2020) with a star tree of the five genomes as input: ((Rhyacophila_brunnea, Himalopsyche_kuldschensis, Himalopsyche_phryganea, Himalopsyche_japonica, Himalopsyche_tibetana)mr).

We used HALtools (Hickey et al., 2013) to obtain global alignment information with the halStats option. We then used “halSummarizeMutations” to generate mutation statistics, including the length of each mutation and the number of substitutions, transitions, transversions, gaps, insertions, deletions, inversions, duplications, and transpositions among genomes.

### 2.6 Population genomic analyses

#### 2.6.1 Taxon sampling

We conducted whole genome resequencing on a total of 46 individuals from four *H. tibetana* populations and four *H. digitata* populations, with six individuals from each population except for pop 1 of *H. digitata*, which included five individuals. All 46 samples were collected as larvae in April 2018 and March 2019 in Nepal. All four *H. digitata* populations and two populations of *H. tibetana* were collected in the headwaters of the Gandaki basin with two additional populations of *H. tibetana* collected in the headwaters of Koshi basin (Figure 6). Since it is presently not possible to identify *Himalopsyche* larvae to species level solely based on morphological characters, we ensured correct identification of these samples based on two molecular markers: the mitochondrial COI and the nuclear CAD, using the methods outlined in Hjalmarsson et al. (2018).

All the samples were preserved in 95% ethanol and archived in the collections of the Senckenberg Research Institute and Natural History Museum (SMF). Specimen and voucher information is shown in Appendix S2 and Figure 6.

#### 2.6.2 DNA extraction, library preparation, and sequencing

Genomic DNA was extracted following a modified salting‐out protocol adapted from Miller et al. (1988), as described in Heckenhauer et al. (2019). In case of low purity DNA (A260/A230 purity ratio below 1.4 and DNA concentration higher than 100 μg/μl that was measured by DeNovix DS11 spectrophotometer), the template DNA was subject to an additional cleanup using magnetic beads as described in Appendix S1 (Section 2.1). Afterward, all the samples were sent to Novogene Co., Ltd. for DNA library preparation and sequencing. 150‐bp paired‐end reads were generated on an Illumina HiSeq 2000 platform.

#### 2.6.3 Quality control, data processing of population sequence data, and variant calling

We assessed the quality of Illumina reads before and after each step using FastQC v0.11.8 (https://www.bioinformatics.babraham.ac.uk/projects/fastqc/) and MultiQC v1.7 (Ewels et al., 2016), details are described in Appendix S1 (Section 2.2). We trimmed overrepresented *k*‐*mers* using autotrim.pl v0.6.1 (Waldvogel et al., 2018) with Trimmomatic v0.38 (Bolger et al., 2014), and we removed adapters with Cutadapt v2.23 (Martin, 2011). After quality filtering, the average sequence depth was 18× and the minimal depth was 12.5× for all the samples. To maintain even depth of all the individuals and to simulate subsets with varying depth levels, we chose 12.5×, 7.5× and 3.5× as our test depth levels. We randomly subsampled the sequencing reads to a specified depth using rasusa 0.3.0. (Hall, 2022) with a random seed of 1 (−s 1), the target depth (−‐coverage 12.5, −‐coverage 7.5, −‐coverage 3.5) and the estimated genome size (−‐genome‐size 550 m) based on GenomeScope2 estimation (see below; Ranallo‐Benavidez et al., 2020; Vurture et al., 2017).

For population genomic analyses, FASTA files of the reference genomes were indexed with the function bowtie2‐build of bowtie2 2.3.5 (Langmead & Salzberg, 2012). We then mapped the reads of the three different depth datasets (12.5×, 7.5× and 3.5×) of the *H. digitata* and *H. tibetana* populations to each of the five different reference genomes separately using Bowtie2, which resulted in 30 datasets (2 species × 3 depth × 5 reference genome, Figure 1). After marking duplicate reads for each dataset using Picard v2.20.8 (Picard Tools – By Broad Institute), we conducted variant calling for downstream population genetic analysis using two strategies. In the first strategy, we called the haplotype of each individual separately, by running GATK v4.1.7.0 (GATK, broadinstitute.org) HaplotypeCaller on each bam file. Then, we called genotypes with GATK GenotypeGVCFs across all resulting vcf files. Ultimately, we selected the variants and filtered missing sites using VCFtools v0.1.17 (Danecek et al., 2011). With the second strategy, we estimated genotype likelihoods with appropriate filtering (including reads/sites/alleles/depth filtering, nonmissing individual, and SNP filtering) using ANGSD v0.931 (Korneliussen et al., 2014). For details and parameters used, see Appendix S1 (Sections 2.3 and 2.4).

#### 2.6.4 Genetic diversity and population structure analysis

The variants called by GATK with genotype calling were used to estimate π, individual *F*, and pairwise *F*_ST_ (Weir & Cockerham, 1984). These are commonly used measurements for population genetics. *F* is used to directly quantify the alleles inherited from common ancestors in an individual's lineage, π is an index for population‐level genetic diversity quantification, and *F*_ST_ provides a primary description of population genetic differentiation. In this study, π and *F*_ST_ were calculated using VCFtools with a 50‐kb window size. To better understand *F*_ST_ within different datasets (species × reference genome × depth), we applied pairwise *F*_ST_ estimates (pairwise populations among the four populations) and global *F*_ST_ estimates (including all the four populations).

We used the genotype likelihoods estimated by ANGSD to generate a PCA with PCAngsd (Meisner & Albrechtsen, 2018) and individual admixture proportions estimating using NgsAdmix (Skotte et al., 2013) after Linkage pruning with ngsLD (Fox et al., 2019). Further details about tools, parameters, and commands were described in Appendix S1 (Section 2.4). Most plots were generated in Rstudio v3.6.1 (http://www.rstudio.org) (scripts for plotting were included in Appendix S1 (Section 3)).

## 3 RESULTS

### 3.1 New genomic resources

**FIGURE 2:**
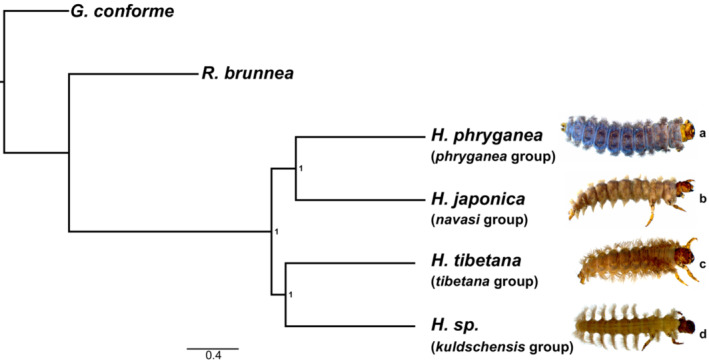
Astral tree of the five reference genomes generated from BUSCO genes. Numbers on the nodes indicate local posterior probabilities. *G. conforme* was used as an outgroup. Images taken from Hjalmarsson et al. (2019), (a) *H. phryganea*, (b) *H. japonica*, (c) *H. gregoryi* (identical with *H. tibetana* morphologically), (d) *H. sylvicola* (identical with *H*. sp. (*kuldschensis* group) morphologically).

**FIGURE 3:**
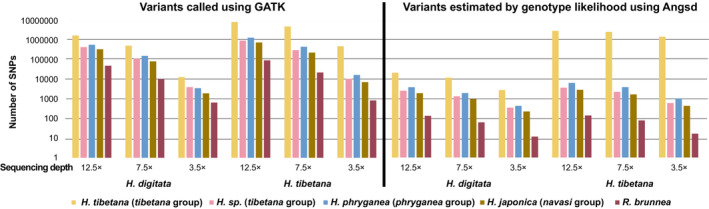
Number of variants called/estimated by two different strategies. Numbers of the Y‐axis were logarithmically scaled.

We combined long‐ and short‐read sequencing technologies to generate three new de novo genome assemblies for the genus *Himalopsyche* (Rhyacophilidae): *H. japonica*, *H*. sp. (*kuldschensis* group), and *H. tibetana*. For each species, we obtained ~150–200× Illumina and ∼18–26× Oxford Nanopore sequencing depth. All three assemblies are of high quality with respect to the number of contigs and contig N50 (Table 1). We identified >96% of the Endopterygota BUSCO gene set in the assemblies. BlobTools detected no contamination (Appendix S1: Figures 1–3). Remapping the Illumina reads back to the assemblies revealed more than 98% could be unambiguously placed (Appendix S1: Figures 1–3).

**FIGURE 4:**
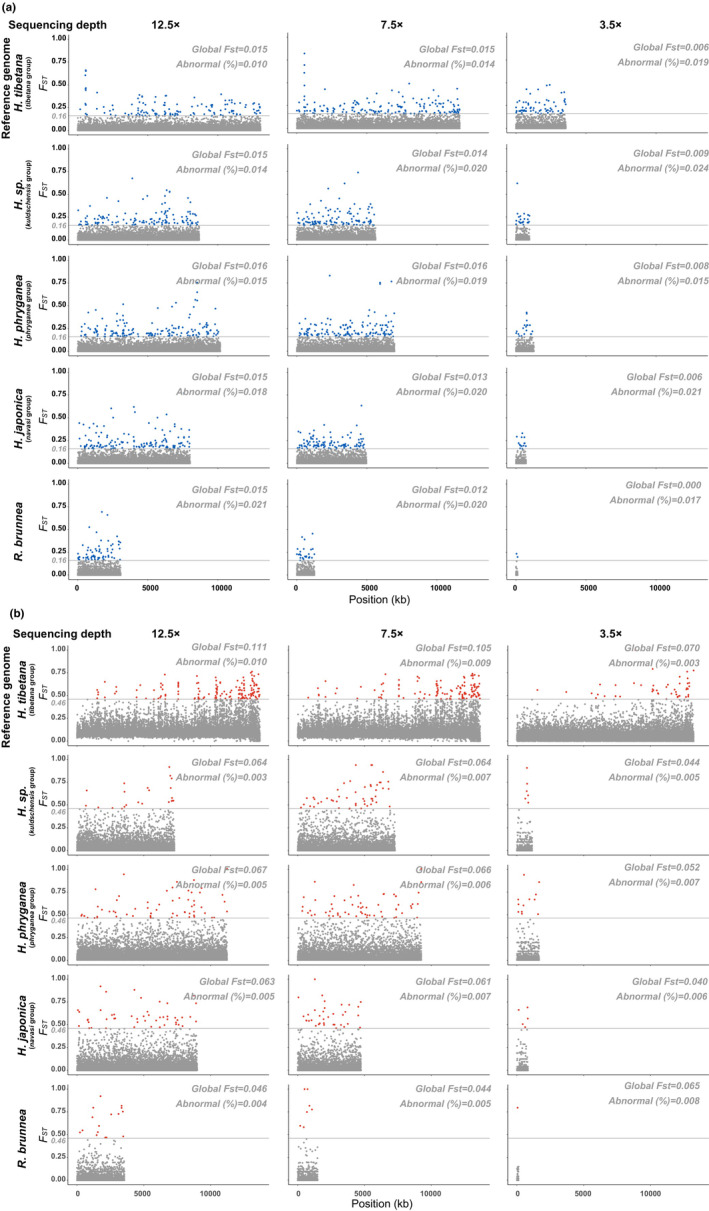
Genome‐wide distribution of *F*_ST_ values (weighted) of (a) the *H. digitata* populations and (b) *H. tibetana* populations. *F*_ST_ values were calculated in 50‐kb windows across contigs obtained from each reference genome; thus, the X‐axis (position) is not comparable among datasets. The horizontal gray lines indicate a threshold that is used for selecting the abnormal *F*_ST_ windows for each species. The threshold is the minimum value of the top 1% *F*_ST_ windows on the whole genome when using *H. tibetana* as reference genome and 12.5× as sequencing depth (0.16 for *H. digitata* and 0.28 for *H. tibetana*). The percentage of abnormal *F*_ST_ windows is the number of *F*_ST_ windows above the threshold over the total number of *F*_ST_ windows. The global *F*_ST_ is the genome‐wide weighted *F*st value estimated based on Weir and Cockerham (1984).

**FIGURE 5:**
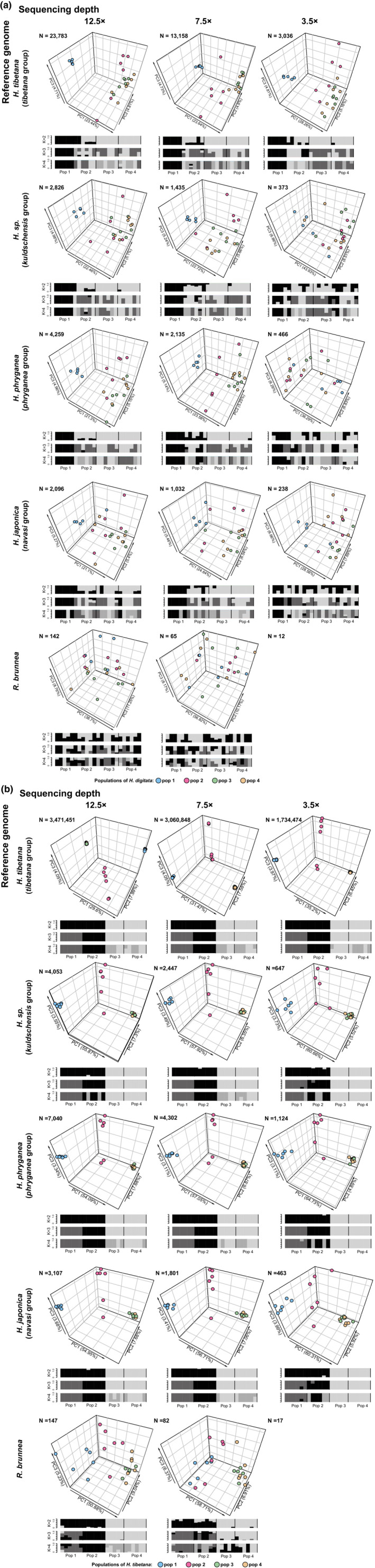
3D scatter plots of all individuals derived from PCA by using *PCAngsd* and population structure (*k* = 2, 3, 4, respectively) inferred from *NgsAdmix* depending on different reference genomes and different sequencing depth of (a) *H. digitata* and (b) *H. tibetana* populations. The explained variances are shown as percentages. Numbers on the top left of each plot show the number of SNPs used for the structure estimating. PCA and admixture were not able estimate when using *R. brunnea* as reference genome with 3.5× depth due to the limited number of variants. Colors in the plots represent the four populations of *H. digitata* and *H. tibetana*, respectively.

**FIGURE 6:**
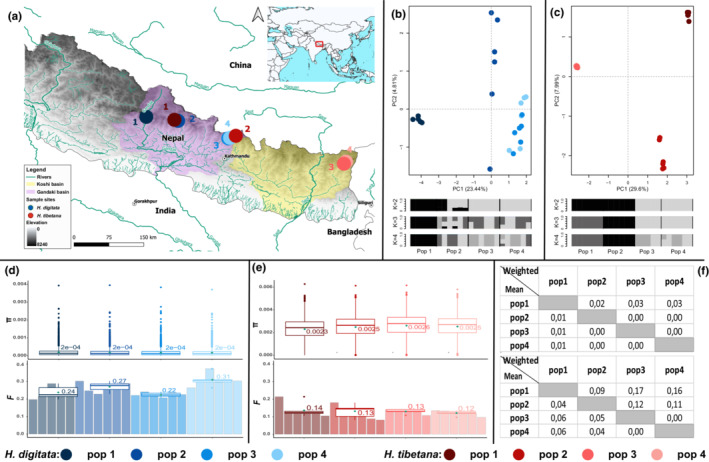
Location of populations, genetic diversity, and population structure of *H. digitata* and *H. tibetana*. (a) Map showing the eight population sites of the two species across two main drainage basins in Nepal. Population numbers are signed on the sample sites; (b) PCA plots and admixture proportions (*k* = 2, 3, 4) of *H. digitata*; (c) PCA plots and admixture proportions (*k* = 2, 3, 4) of *H. tibetana*; (d) nucleotide diversity (upper) and inbreeding coefficient (lower) of *H. digitata*, the mean values of each population were labeled with green dots and numbers; (e) nucleotide diversity (upper) and inbreeding coefficient (lower) of *H. tibetana*, the mean values of each population were labeled with green dots and numbers; (f) pairwise *F*_ST_ of *H. digitata* (upper) and *H. tibetana*.

The estimated genome sizes resulting from the *k*‐*mer*‐based estimation with Genomescope2 were 481 Mb (*H. japonica*), 495 Mb (*H*. sp. (*kuldschensis* group)), and 568 Mb (*H. tibetana*; Appendix S1: Figures 4–6). Between 31% (*H. japonica*) and 44% (*H. tibetana*) of the genome assemblies were identified as repeats. A high percentage of the repeats were classified as interspersed repeats (approx. 28.5–40.3%). More than half of the interspersed repeats remain unclassified and therefore may be specific to Trichoptera. Details on repeat classes are given in Appendix S1 (Tables S1–S3). The annotation of the genomes resulted in the prediction of 9983 (*H. japonica*), 10,049 (*H*. sp. (*kuldschensis* group)), and 10,994 (*H. tibetana*) proteins. Most of the annotated proteins had functional Blast2GO annotations, were verified by BLAST, or were mapped to GO terms. GO Distributions were similar to previously annotated caddisfly genomes, that is, the major biological processes were cellular processes. Catalytic activity was the largest subcategory in molecular function, and the cell membrane subcategories were the largest cellular component (Appendix S1: Figures 7–12).

The Cactus alignment of the five reference genomes showed that *Rhyacophila brunnea* had the longest sequence length and largest number of contigs (Appendix S4). It also showed a higher level of mutations compared with the four *Himalopsyche* genomes, for instance gaps, insertions, inversions, and duplications. The number of mutations among the four *Himalopsyche* genomes was at similar levels in the Cactus alignment over all.

### 3.2 Phylogenetic relationships of the reference genomes—resolving the *Himalopsyche* backbone

We built a species tree for the five reference genomes to estimate their evolutionary history and to verify the phylogenetic relationship between the reference genomes and the two population‐level target species, *H. digitata* and *H. tibetana*. Phylogenetic relationships were strongly supported (Figure 2). Our phylogenetic tree showed two pairs of sister species (*H. tibetana* + *H*. sp. (*kuldschensis* group); *H. phryganea* + *H. japonica*) with *R. brunnea* forming the sister clade of all four *Himalopsyche* species. According to Hjalmarsson et al. (2018, 2019), *H. digitata* and *H. tibetana* both belong to the *tibetana* group, *H*. sp. to the *kuldschensis* group, *H. phryganea* to the *phryganea* group and *H. japonica* to the *navasi* group. In contrast to our results, Hjalmarsson et al., 2019 recovered the *navasi* group sister to all other Himalopsyche and a sister relationship between the *phryganea* and *kuldschensis* groups. Differences could be related to the different sampling strategies. Here, we present much more genome‐wide data per taxon, but reduced taxon sampling. For the purpose of assessing the impact of reference genome relatedness in SNP calling, *H. tibetana* and *H. digitata* (both belonging to *tibetana* group) are most closely related to *H. tibetana*, followed by *H*. sp. (*kuldschensis* group), *H. phryganea* and *H. japonica*, with *R. brunnea* being the most distantly related species.

### 3.3 The impacts of sequencing depth and phylogenetic relatedness of the reference genome on population genetic studies

To better understand the impacts of sequencing depth and reference genome on population genomic analyses, we compared the number of quality‐filtered variants, genetic diversity and population structure of the species *H. digitata* and *H. tibetana* among the datasets based on different reference genomes and varied sequencing depth. The results revealed that, perhaps unsurprisingly, the most distantly related reference genome, or the lowest sequencing depth, resulted in the least accurate downstream analysis.

We observed that reference genome selection has a measurable impact on the number of variants (Figure 3). With both strategies (variant calling using GATK and genotype likelihood estimation using Angsd), the number of variants sharply decreased when the reference genome was more distantly related, but remained similar with decreasing sequencing depth, especially when estimated by genotype likelihood. This was particularly striking for the *H. tibetana* populations when using ANGSD to call the variants, which dropped from millions to thousands when changing the reference genome from *H. tibetana* to the others (Figure 3). Consequently, the massive loss of information resulting from choosing a distant reference genome is likely to lead to poor performance in the downstream analyses.

We observed that the choice of reference genome influenced population genetic values such as pairwise *F*_ST_ (Appendix S3) and nucleotide diversity estimates (Appendix S1: Figure 14), the number of outliers in the inbreeding coefficient estimates (Appendix S1: Figure 13), as well as the resolution of the final outcome in genome‐wide *F*_ST_ estimates (Figure 4), PCA, and admixture (Figure 5). For example, in the case of pairwise *F*_ST_ estimates, when changing the reference genome from *H. tibetana* to *R. brunnea* (with the same sequencing depth, e.g., 12.5×), the *F*_ST_ value between pop 1 and pop 3 of *H. tibetana* reduced from 0.17 to 0.06 (Appendix S3). The number of outliers (*F* < 0) in inbreeding coefficient estimates increased when using a reference genome that is distantly related to the target species, while the inbreeding coefficient values of individuals tended to fluctuate for both species, which might result in an ineffective or misleading conclusion (Appendix S1: Figure 13). The results of the PCA, admixture analysis, and the genome‐wide *F*_ST_ clearly showed notable decreases in the resolution of the plots when selecting a more distantly related reference genome (Figure 5). For instance, for populations of *H. digitata*, regardless of the depth, the cluster of pop 1 was well defined when mapped to *H. tibetana* in the PCA plot and admixture analysis, but no distinguishable structure was discernible among all four populations when mapped to *R. brunnea* (Figure 5a). In addition, using a conspecific reference genome significantly improved the accuracy of genome‐wide *F*_ST_ estimates, including both the global *F*_ST_ value (the global *F*_ST_ value of *H. tibetana* populations doubled when mapped to *H. tibetana*) and the distribution of *F*_ST_ values from sliding windows. This effect was particularly pronounced with the abnormal value (highly qualified resolution, Figure 5b).

We also observed extensive influence of sequencing depth on the downstream analyses. In addition to decreasing number of variants, low sequencing depth also affected estimates of nucleotide diversity, the inbreeding coefficient, pairwise and global *F*_ST_, and population structure. In most cases, low sequencing depth reduced the accuracy of these inferences; however, the influence proved to be negligible or limited in some treatments. For example, the population structure of *H. tibetana* was highly differentiated regardless of depth when mapped to *H. tibetana* and was less differentiated when sequencing depth decreased while mapped to *H. phryganea*, but still sufficient to obtain a reliable result (Figure 5b). Likewise, the *F* value of all individuals changed when the sequencing depth decreased from 12.5× to 3.5×, but the differentiation among populations remained comparable (Appendix S1: Figure 13).

Furthermore, the results revealed that reference genome and sequencing depth had variable impacts between the populations of *H. digitata* and *H. tibetana*. More specifically, the same treatment (same reference genome and sequencing depth) might be sufficient to generate a reliable result for the populations of *H. tibetana*, but not for *H. digitata*. For instance, when mapped to *H. japonica*, the population structure of the *H. tibetana* populations was distinguishable regardless of sequencing depth, but it was barely detectable for the *H. digitata* populations even with 12.5× depth (Figure 5). Considering the inherent genetic variation of the two species, it is not surprising that more distantly related reference genome or lower sequencing depth were more tolerable for the populations with higher genetic diversity.

### 3.4 Genetic diversity and population structure of *H. digitata* and *H. tibetana*

Each population of *H. digitata* had a similar level of nucleotide diversity, but less consistent *F* values: pop 4 was slightly higher (0.31), whereas the other three were similar (0.22–0.27, Figure 6d). Population 1, which is located in the main river of Gandaki basin in central western Nepal, formed a distinct cluster in the PCA analyses (Figure 6a,b). Moreover, no admixture signal was detected in pop 1. As a population located in the middle of the basin, but more closely connected with pop3 and pop 4 by catchment, pop 2 formed a distinct cluster and adjoined pop3 and pop4 in the PCA analysis. Meanwhile, pop 2 was represented as genetic mixtures regardless of *K* values. Population 3 and 4 were mixed together in the PCA analyses, which was in line with the fact that they were located closely together in the most northeastern tributaries of the Gandaki basin. In addition, pop 3 and pop 4 were homogeneous when *K* = 2, but mixed when *K* = 3 or 4. This population structure was further supported by the pairwise *F*_ST_ estimates using the base‐called variants (Figure 6f).

Following the pattern of *H. digitata*, the populations of *H. tibetana* had a very similar level of both nucleotide diversity and inbreeding coefficient (Figure 6e) and a more distinct population structure congruent with catchments (Figure 6c,f). Moreover, compared with *H. digitata*, *H. tibetana* showed a greater population diversity among the four populations, including higher nucleotide diversity (~ 12‐fold) and *F*_ST_ (both pairwise and globalwise, Figures 6f and 4), lower inbreeding coefficient (~ 0.5‐fold), as well as a more distinct population structure (Figure 6b,c). This is in accordance with the fact that the geographic locality and catchment connection of the *H. tibetana* populations were further apart from each other compared with the ones of the *H. digitata* populations.

## 4 DISCUSSION

### 4.1 Reference genomes

In terms of BUSCO completeness and contiguity, the three de novo genomes provided in this study as references are of comparable quality than the other Trichoptera genomes published previously (Heckenhauer et al., 2019, 2022; Luo et al., 2018; Olsen et al., 2021; Ríos‐Touma et al., 2022). They contained 96%–97% of an Endopterygota core gene collection indicating an almost complete coverage of known single‐copy orthologs in the assembly. The backmapping rate of Illumina reads to the assemblies ranged between 96 and 98%, which also indicates the high quality of the assemblies.

Previous genomic studies have focused on sequencing a wide range of different families of Trichoptera and investigating variations across the order (Heckenhauer et al., 2022). These three new genome assemblies provide important new genomic resources for the scientific community, especially since Trichoptera and other aquatic insects are in general underrepresented in genomic research (Hotaling et al., 2020, 2021). Together with previously published ones (*H. phryganea* and *R. brunnea*), these newly available genomes adequately provide an initial perspective about the phylogenetic relationship of the four main taxonomic groups of *Himalopsyche*, as well as implying the genetic relatedness between the target species and the five different reference genomes, respectively, for this study.

### 4.2 Impacts of reference genome and sequencing depth on population genetic inferences

High‐throughput sequencing of individual specimens is poised to become the state‐of‐the‐art in population genetics studies. However, despite falling prices in sequencing, this approach can be prohibitively costly for large number of samples in species with large genomes. Many researchers may thus face the choice of either using an existing nonconspecific reference genome or lowering sequencing depth of individual samples. Previous studies have demonstrated that either the reference genome or the sequencing depth has a direct influence on population genetic estimates (Garcia‐Rubio et al., 2018; Gopalakrishnan et al., 2017; Valiente‐Mullor et al., 2021; Yang et al., 2019). Studies assessing the joint effects of the reference genome and sequencing depth are rare and hitherto lacking in insects. Here, we evaluated population diversity and structure based on a range of reference genomes and varied levels of low sequencing depth in the first case study on insects. The results revealed that the choice of the reference genome and sequencing depth both had an influence on estimating population genetic indices, including *F*, π and *F*_ST_, as well as the genetic structure inferred from genotype likelihoods. To some extent, the general trends are that (a) the more closely related the reference genome is, the more stable the estimates of population genetic indices are, and (b) the higher the sequencing depth, the better the resolution of the population structure analyses. However, the results also vary depending on the inherent genetic variation of the target species.

The choice of reference genome has a stronger influence on downstream analyses than resequencing depth, which is consistent with previous studies (Garcia‐Rubio et al., 2018; Günther & Nettelblad, 2019; Valiente‐Mullor et al., 2021). This is mainly due to the massive loss of reads while mapping to a distantly related reference genome. For example, in *H. tibetana*, mapping rates decrease from ~98% when using a conspecific reference genome, to 10% with a reference genome of a species of another genus (*Rhyacophila brunnea*; Appendix S5). This leads to a dramatic decline of variants (Figure 3). Moreover, increasing mismatches may also occur due to alternative alleles, thus increasing the so‐called “reference bias” (Günther & Nettelblad, 2019). Consequently, reference bias can impact variant calling by missing alternative alleles or by incorrectly calling heterozygous sites and therefore lead to an underestimation of variants, including rare/private variants (Günther & Nettelblad, 2019; Taub et al., 2010). Considering these two aspects, the effects related to the choice of reference genome may propagate to a certain degree to subsequent downstream analyses in a population genetic study, for example when investigating heterozygosity and genetic diversity, gene flow, as well as ancestry proportions (Brandt et al., 2015; Günther & Nettelblad, 2019). We have observed these in our results: the number of variants decreases logarithmically with genetic relatedness of the reference genome, especially for the genotype likelihood dataset called by ANGSD, which includes a strict filtering on base level, read level, sequencing depth, and other levels during the genotype calling. Moreover, when the genetic relatedness between reference genomes and target species decreases, the population genetic indices including *F*, π, and pairwise *F*_ST_ tend to become less accurate, especially at a low depth (3.5×). In addition, estimates of population structure, including PCA and admixture, are strongly affected, resulting in unreliable, weakly supported findings.

Notably, even though population genetic analyses of both *H. tibetana* and *H. digitata* show the greatest accuracy when using *H. tibetana* as the reference genome, we have observed a remarkable improvement in the results when using a conspecific reference genome, for instance, in the high resolution of the population structure, especially with the low sequencing depth (Figure 5b). For example, when mapping *H. tibetana* population samples to the *H. tibetana* reference genome, we observed a mapping rate of 98%. However, mapping success decreases rapidly with decreased relatedness. When mapping *H. digitata* to *H. tibetana*, the mapping rate is only 35% and decreases to 20% when mapped to *H*. sp. *kuldschensis* and 18% when mapped to *H. phryganea*. As a consequence, even though the genome size of the two species is similar (Table 1), the number of variants called from the *H. digitata* population dataset is much lower than variants called from the *H. tibetana* population dataset, especially when estimated with genotype likelihood methods (Figure 3). Although *H. digitata* and *H. tibetana* are recovered in the same clade in previous phylogenetic analyses (Hjalmarsson et al., 2019), the poor mapping success suggests that genetic distance may still be high. Unfortunately, there are no established divergence times within the genus of *himalopsyche* at present. The most recent study (Thomas et al., 2020) shows that the divergence between *Rhyacophila* and *Himalopsyche* was approx. 90 Ma. Considering the distinct ecological niches of *H. tibetana* (inhabits high altitude) and *H. digitata* (inhabits a lower altitude) in the same distribution range (both endemic to the Himalayas), we hypothesize that the divergence between these two species may be associated with the uplift of the Qinghai–Tibet Plateau, which began ca. 45 Ma ago (Ding et al., 2022). In summary, considering the long evolutionary history of caddisflies in general (ca. 280 Ma), and the rapid radiation of caddisflies (Thomas et al., 2020), a high level of divergence between two closely related caddisflies species is not entirely unexpected. Therefore, we suggest that when selecting a closely related species as a reference genome in a population genomics study, it is important to consider genetic relatedness.

We also show that sequencing depth must be considered when designing population genomic analyses. Unlike de novo genome assembly which demands high sequencing depth, highly accurate results can be achieved with lower sequencing depth in population genomics by combining information from a large number of individuals either during SNP calling or other processes (Buerkle & Gompert, 2013; Fumagalli, 2013; Han et al., 2014). Even though the accuracy of population genetic inferences can be improved by increasing the number of samples, the bias caused by low sequencing depth cannot be ignored, especially since it can often be difficult to obtain many samples for some populations of rare animals. As revealed by previous studies, low sequencing depth may cause erroneous SNP calls, due to the errors introduced and amplified during PCR during library prep (Sims et al., 2014). Moreover, it may also produce ambiguous reads during mapping to the reference genome (Taub et al., 2010). Such biases may lead to incorrect conclusions in population genetics inferences, including population genetic differentiation, population structure, and demography (Crawford & Lazzaro, 2012; Fumagalli, 2013; Han et al., 2014; Jiang et al., 2019; Korneliussen et al., 2013). Our results show that decreasing depth massively reduced the number of informative sites, which may largely result from the variant calling step, especially when applying a series of filtering approaches, thus leading to a less accurate result in downstream analyses. However, sequencing depth may have limited impact in some other cases: The results are not affected by depth when the reference genome is either very closely or very distantly related to the target species. For example, when using *H. tibetana* as reference genome with *H. digitata* (the most closely related reference), population structures are distinguishable regardless of sequencing depth (Figure 5a). However, when using *H. japonica* or *R. brunnea* as the reference genome (most distantly related), population structures are indistinguishable regardless of the sequencing depth. Therefore, increasing the sequencing depth may not improve the results in such cases where the reference genome is too distantly related.

To conclude, both reference genome and sequencing depth have various degrees of influence on downstream analyses, whereas their respective impact is different for each target species. Our results imply that populations with a higher genetic diversity are less affected by the relatedness of the reference genome and the sequencing depth in population structure analyses. In general, the results for *H. tibetana* appeared more robust despite the variation of reference genome and sequencing depth in comparison to those obtained for *H. digitata*. This may result from (1) different pairwise relatedness between reference genome and target species and (2) inherent population variation (or expected level of differentiation). Even though *H. tibetana* and *H. digitata* are very closely related and both belong to the *tibetana* group (Hjalmarsson et al., 2019), populations of *H. tibetana* show a higher level of population variation compared to those of *H. digitata*, as, for instance, shown by higher *F*_ST_ value and nucleotide diversity of populations. This is likely caused by different distribution patterns with *H. tibetana* populations being more isolated at high elevations than *H. digitata*. A detailed investigation of this was not intended with this study, and our sampling is insufficient and thus inconclusive regarding the underlying biological reasons for the differences in species‐specific population structures.

Similar to the trade‐off between sequencing depth and sample size demonstrated by previous studies (Buerkle & Gompert, 2013; Fumagalli, 2013), we reveal that the roles of reference genome and sequencing depth in a study of population genetics could also be considered as a trade‐off. We suggest that population genetic study using genomic data may benefit from applying a more closely related reference genome. Undoubtedly, the optimal option would be a conspecific reference genome even with a low sequencing depth. Our results showed that a conspecific reference genome can significantly improve the accuracy and reliability of all kinds of analyses, in particular WGR‐based SNP imputation which will be promising in other genomic investigations like genome‐wide association studies. However, if resource limitations exist (in terms of funding, available biological material, time (i.e., wet‐ and dry lab efforts)), or the research is focused on populations with high interpopulation variation, a trade‐off between a more distant reference genome and a higher sequencing depth can be considered. In other words, in a population genetic study, the trade‐off between reference genome and sequencing depth is dictated by the focus of research. Although our case study is carried out on caddisflies and thus may not be universally applicable to other organisms, we believe that our results do provide a valuable example that enhances developing roadmaps involved in the choice of appropriate reference genome and sequencing depth in population genomic studies.

### 4.3 Concordance between genetic patterns and biogeography of *H. digitata* and *H. tibetana*

Irrespective of sequencing depth and reference genome, the results of the population genetic analyses for *H. tibetana* and *H. tibetana* are highly consistent with the geographic distribution of populations within the drainage and river networks. For example, in *H. tibetana*, the PCA plots show that pop1 and pop 2 form two distinct clusters, respectively, while pop 3 and pop 4 cluster together and are separated from pop1 and pop 2. This is also consistent with their geographical location: pop 3 and pop 4 are both located in the Kanchenjunga region in far eastern Nepal; pop 2 is located in the most northeastern tributaries of the Gandaki Basin, and pop 1 is located in the east of the Annapurna circuit, which is in the center of the Gandaki Basin. In the admixture plots, pop 1 and 2, as well as pop 3 and 4, share the same structure, respectively. These results are consistent with the basin structure of these populations (pop 1 and pop 2 in Gandaki basin; pop 3 and pop 4 in Koshi basin). Moreover, compared with *H. digitata* populations, which are all located in one basin, *H. tibetana* populations show greater genetic diversity and clearer population structure. Due to the dependency of larvae on the aquatic habitat (De Moor & Ivanov, 2007), the limited dispersal capabilities of the adults (Griffith et al., 1998; Petersen et al., 2004), and the unique geographic feature of the Himalayan region, it is not surprising that the population genetics of the two target species show a notable correlation with drainages and river network, like in other Trichoptera studied in the region (Hoppeler et al., 2016) and elsewhere (Altermatt et al., 2013; Engelhardt et al., 2011). The resequencing approach, even with low sequencing depth, appears to be a suitable methodological avenue to study population genomics of insect populations when reference genomes of at least moderate relatedness are available.

## AUTHOR CONTRIBUTIONS

**Xiling Deng:** Data curation (equal); formal analysis (equal); methodology (equal); software (equal); validation (equal); visualization (equal); writing – original draft (equal); writing – review and editing (equal). **Paul B. Frandsen:** Conceptualization (equal); data curation (equal); formal analysis (equal); investigation (equal); methodology (equal); resources (equal); software (equal); supervision (equal); validation (equal); writing – review and editing (equal). **Rebecca B Dikow:** Data curation (equal); methodology (equal); resources (equal); software (equal); writing – review and editing (equal). **Adrien Favre:** Writing – review and editing (equal). **Deep Narayan Shah:** Investigation (equal); resources (equal); writing – review and editing (equal). **Ram Devi Tachamo Shah:** Data curation (equal); resources (equal); writing – review and editing (equal). **Julio V. Schneider:** Data curation (equal); methodology (equal); resources (equal); writing – review and editing (equal). **Jacqueline Heckenhauer:** Conceptualization (equal); data curation (equal); formal analysis (equal); investigation (equal); methodology (equal); resources (equal); software (equal); supervision (equal); validation (equal); visualization (equal); writing – original draft (equal); writing – review and editing (equal). **Steffen Pauls:** Conceptualization (equal); data curation (equal); funding acquisition (equal); investigation (equal); project administration (lead); resources (equal); supervision (equal); validation (equal); writing – review and editing (equal).

## CONFLICT OF INTEREST

None declared.

## Supporting information


Appendix S1

Appendix S2

Appendix S3

Appendix S4

Appendix S5

## Data Availability

All the COI and CAD data for this research are available in the GenBank of National Center for Biotechnology Information (NCBI), and the accession codes of each individual are provided in Appendix S2. The raw data and genome assemblies of the three novel references have been deposited at NCBI under the Bioproject ID PRJNA728835, and the raw data of populations have been deposited at NCBI under the Bioproject ID PRJNA749154. All BUSCO genes, annotation gffs, and predicted proteins resulting from GEMOMA, blastp, and BLAST2GO results, as well as repeatmodeler and ‐masker results are available at: https://doi.org/10.6084/m9.figshare.c.6033011.v1 [dataset]. The two reference genome of *H. phryganea* and *R. brunnea* were previously published by Heckenhauer et al. (2022) and has been deposited at NCBI under the BioProject ID PRJNA558902.
