# Supplementary material for: The impact of sequencing depth and relatedness of the reference genome in population genomic studies: A case study with two caddisfly species (Trichoptera, Rhyacophilidae, Himalopsyche)
Source: Ecol Evol. 2022 Dec 12;12(12):e9583. doi: 10.1002/ece3.9583 (PMC9745013; doi:10.1002/ece3.9583)
Supplement: Supplementary file 1 — Appendix S1 [file ECE3-12-e9583-s001.docx]

Making a trade-off decision between reference genome and sequencing depth in population genetic studies, a case study with two caddisfly species (Trichoptera, Rhyacophilidae, *Himalopsyche*)

Xi-Ling Deng^1,2,3,*^, Paul B. Frandsen^3,4,5^, Rebecca B. Dikow^5^, Adrien Favre^1,6^, Deep Narayan Shah^7^, Ram Devi Tachamo Shah^8,9^, Julio V. Schneider^1^, Jacqueline Heckenhauer^1,3,+^ Steffen U. Pauls^1,2,3,+^

# 1 *De novo* genomes of three reference species

## 1.1 Sequencing strategies and sequencing read processing

### Illumina short reads sequencing

All three species (*H. tibetana*, *H. sp.* (*kuldschensis* group)*,* *H. japonica*) were subject to Illumina paired-end sequencing. We prepared genomic libraries for Illumina sequencing from 400-500 ng gDNA using the NEBNext Ultra II FS DNA Library Preparation Kit (New England Biolabs, Ipswich, MA, USA) following the manufacturer’s manual. To achieve a mean insert size of 400-500 bp, we conducted enzymatic fragmentation with subsequent size selection. We chose a combinatorial dual indexing approach using the NEBNext Multiplex Oligos for Illumina (Dual Index Set 1) and amplified libraries with five PCR cycles. We quantified each library on a Qubit 4.0 fluorometer with the 1x dsDNA HS Assay Kit and checked the fragment size distribution on a 2200 TapeStation using a High Sensitivity D1000 Tape. Illumina paired-end (150 bp) sequencing was done on a HiSeq 2000 sequencer at Novogene. After checking the quality of Illumina reads using FastQC v0.11.8 (<http://www.bioinformatics.babraham.ac.uk/projects/fastqc>), we trimmed of overrepresented k-mers using autotrim.pl v0.6.1 (Waldvogel et al., 2018) with Trimmomatic v0.38 (Bolger et al., 2014) and a custom adapter file (ILLUMINACLIP: <adapter_combined.fa>:2:30:10), SLIDINGWINDOW:4:20 and MINLEN:50 and further processed reads with Cutadapt v2.23 (Martin, 2011) using the following parameters:-- pair-filter=any -l=140, --max-n=0 for sample which showed a warning flag at per base sequence content in FastQC. To filter out potentially contaminated reads, we used Kraken 2 v2.0.8-beta (Wood et al., 2019; Wood & Salzberg, 2014) with the standard Kraken 2 database and only kept unclassified reads for further analyses.

### Oxford Nanopore sequencing

To generate Oxford Nanopore long reads we sheared high-molecular-weight gDNA (2.0-3.2 µg; > 60 kb) to a mean fragment size of about 10 kb using g-TUBES (Covaris, Woburn, MA, USA), centrifuging the samples at 6000 rpm for 1 min in an Eppendorf 5424 Centrifuge. Libraries were prepared with the SQK-LSK109 kit (Oxford Nanopore, Oxford, UK) according to the manufacturer’s manual (version: GDE_9063_v109_revB_23May2018) with the following modifications: (1.) During DNA repair and end-prep, we used nuclease-free water instead of DNA CS; (2.) the incubation time of the end-prep reaction was 15 min (instead of 5 min) at 65°C; (3.) mixing was always done by pipetting; (4.) we used 80% ethanol (instead of 70%) for magnetic bead-based cleanup; (5.) we increased the time for DNA-binding to the magnetic beads to 15-20 min; (6.) the magnetic beads were air-dried for 1-2 min; (7.) after the adapter ligation, we carried out the clean-up with 45 µl Ampure XP beads (instead of 40 µl; Beckman Coulter, Brea, CA, USA); (8.) when preparing the library for loading onto the flow cell, we used 14 µl (instead of 12 µl) of the DNA library. To enrich for long fragments after adapter ligation, we used the L Fragment Buffer (LFB). After both the end-prep and adapter ligation steps, we quantified DNA using a Qubit 4.0 fluorometer.

We sequenced each Oxford Nanopore library on a single flow cell using the MinION portable DNA sequencer. For basecalling Nanopore reads from their raw .fast5 files, we used Poretools v0.6.0 (Loman & Quinlan, 2014) or Guppy Basecalling Software v2.3.1 (https://nanoporetech.com/nanopore-sequencing-data-analysis). Reads resulting from the whole genome amplification protocol were demultiplexed using guppy_barcoder with --barcode_kits "EXP-NBD104". After trimming adapters with Porechop v.2.0.4 (https://github.com/rrwick/Porechop) using default parameters, we used FASTQ Screen like tools (<https://github.com/schellt/fqs-tools>) to filter out potential contaminants. For this purpose, we mapped the long reads to a custom-made database with minimap2 –x map-ont. The database contains viral, bacteria and human sequences, as well as potential parasites of Trichoptera (Ascogregaria, Gregarina, Mermithidae) and Trichoptera genomes (as positive control). We created an ID list with awk and extracted the screening info with the paf2fqs.pl script of FASTQ Screen like tools (https://github.com/schellt/fqs-tools). Screening info was extracted with the paf2fqs.pl script from FastQ Screen like tools using the paf. file obtained from minimap2 and the previously created ID list. We kept reads without hits and reads that mapped to Trichoptera genomes with subseq command of seqtk 1.3-r106 (https://github.com/lh3/seqtk).

## 1.2 Contamination screening using Bloptools

The final genome assemblies were screened and filtered for potential contaminations with taxon-annotated GC-coverage (equal to depth in this scenario) plots (TAGC plots) plots using BlobTools v1.0 (Laetsch & Blaxter, 2017). For this purpose, all preprocessed Illumina reads were mapped against the final genome assemblies using BWA-MEM v0.7.17-r1188 (Li, 2013). Taxonomic assignment for BlobTools was done with blastn using the following parameters: -*task megablast*, *max_target_seqs 1*, and -*max_hsps 1*. No contaminations were detected in the assemblies.


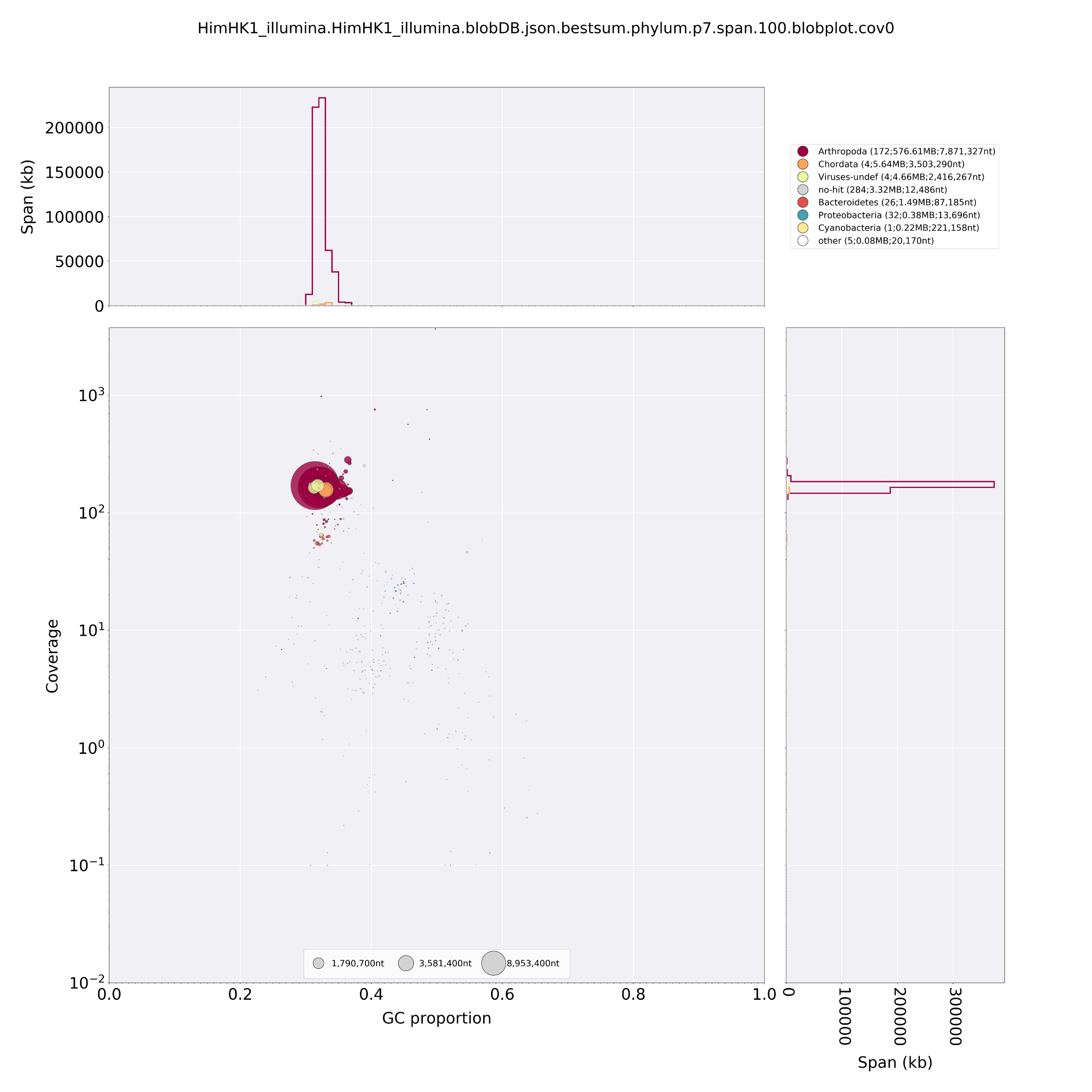


**
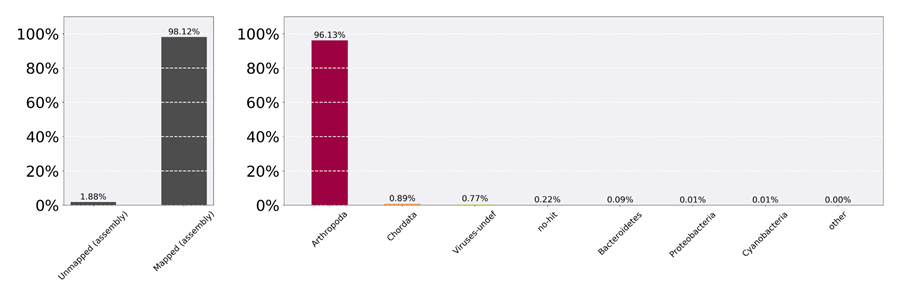
Supplementary Figure 1: Taxon-annotated GC-coverage (TAGC) and ReadCov plots of *H. sp.* (*kuldschensis* group) genome assembly.** Circles indicate contigs and the color indicates the best match to taxon annotation. The upper and right-hand panel show the total span of contigs (kb) given GC proportion. ReadCovPlot visualise the proportion of (un-)mapped reads and the percentage of mapped reads by taxonomic group.


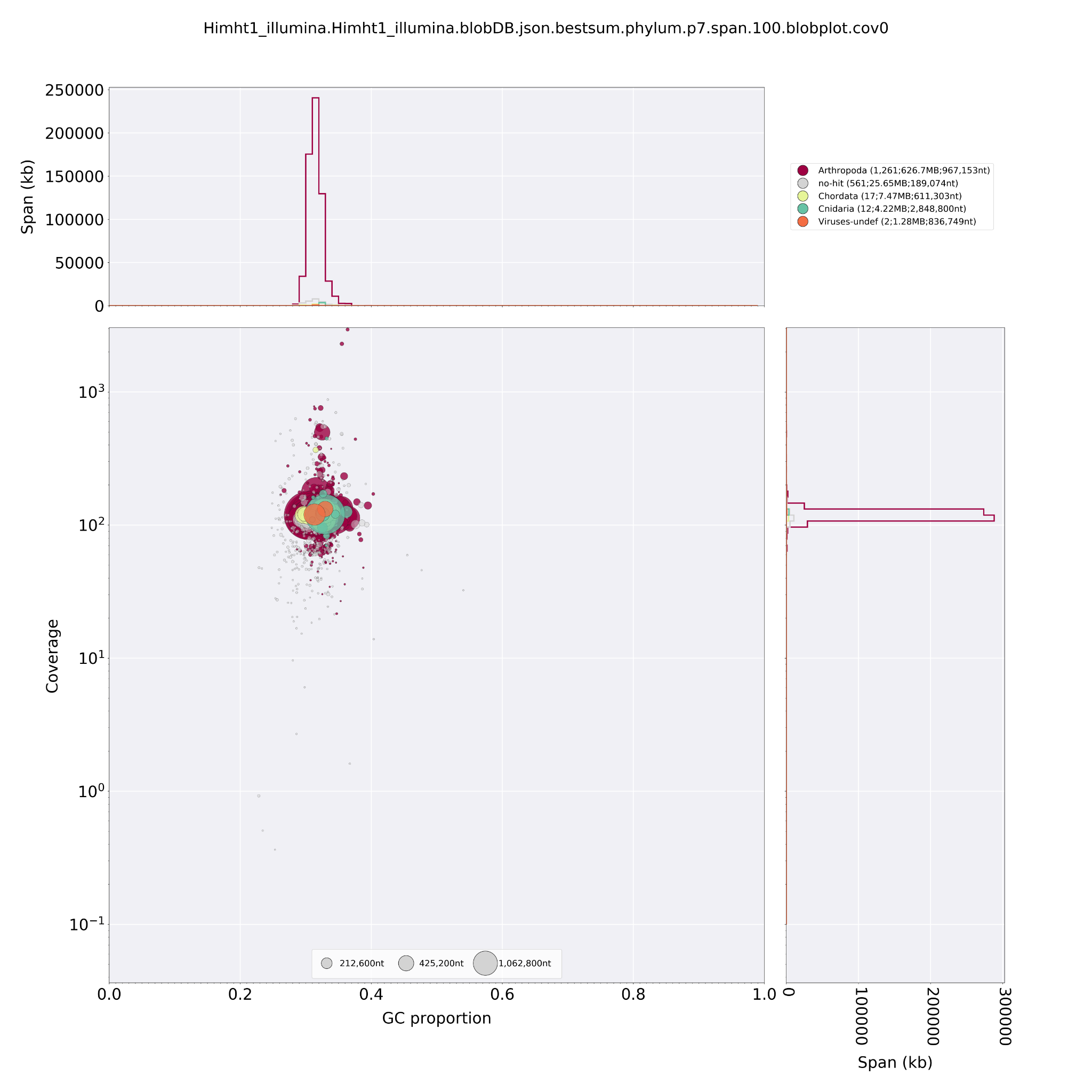


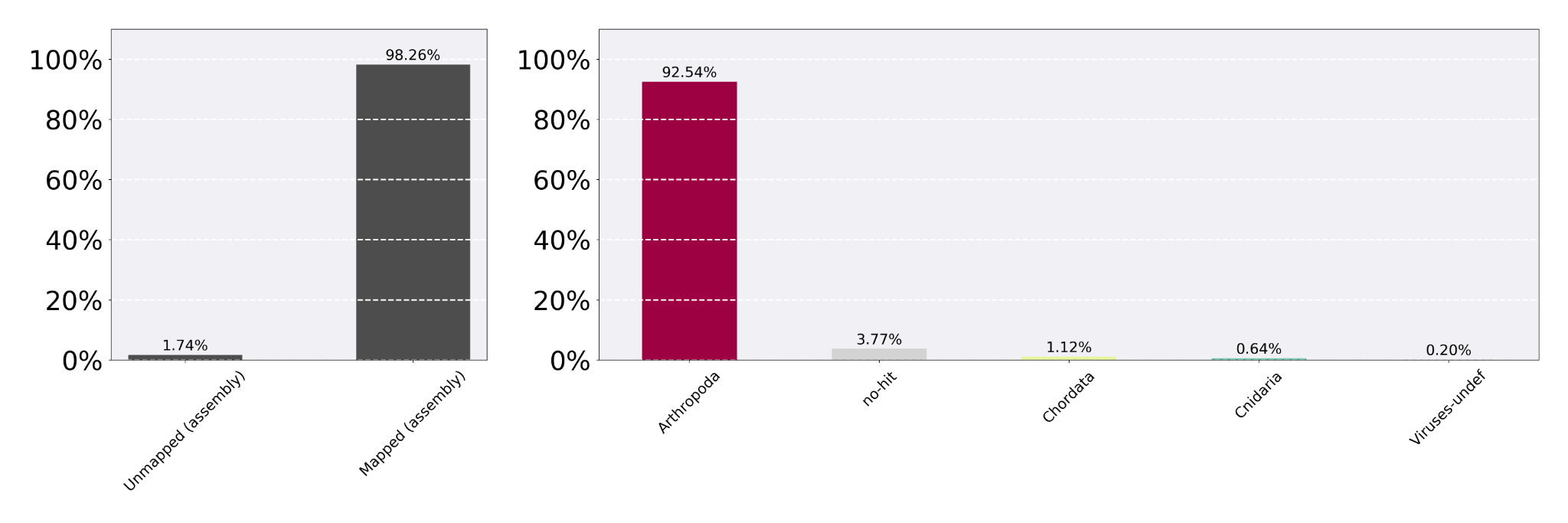


**Supplementary Figure 2: Taxon-annotated GC-coverage (TAGC) and ReadCov plots of *H. tibetana* genome assembly.** Circles indicate contigs and the color indicates the best match to taxon annotation. The upper and right-hand panel show the total span of contigs (kb) given GC proportion. ReadCovPlot visualise the proportion of (un-)mapped reads and the percentage of mapped reads by taxonomic group


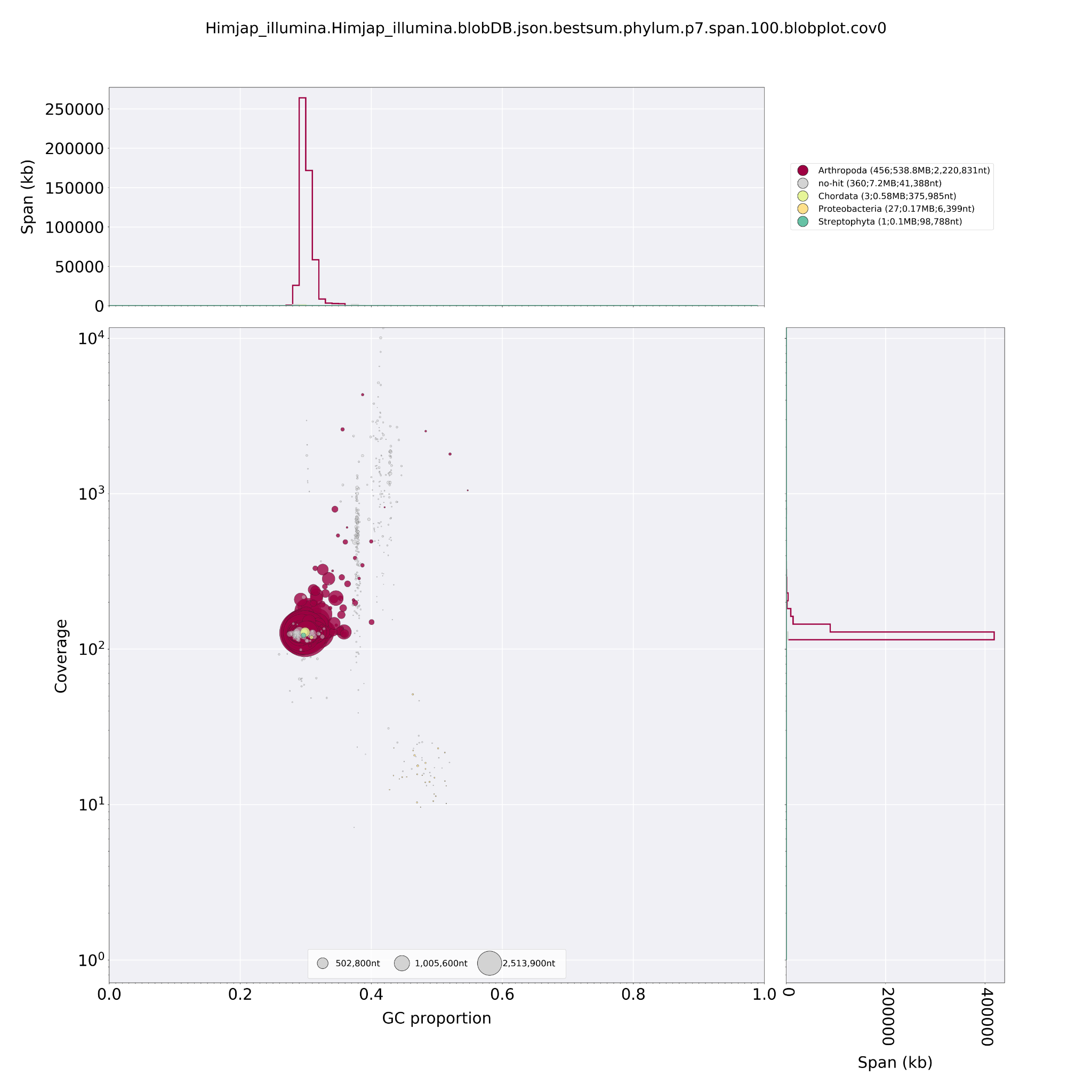


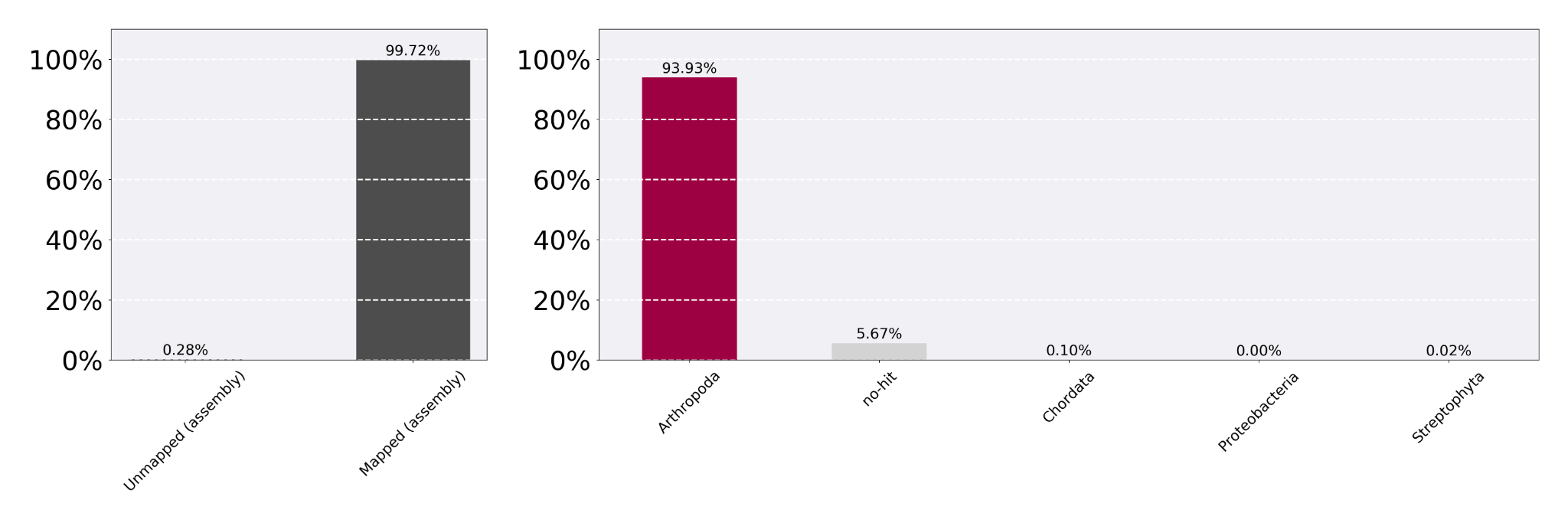


**Supplementary Figure 3: Taxon-annotated GC-coverage (TAGC) and ReadCov plots of *H. japonica* genome assembly.** Circles indicate contigs and the color indicates the best match to taxon annotation. The upper and right-hand panel show the total span of contigs (kb) given GC proportion. ReadCovPlot visualise the proportion of (un-)mapped reads and the percentage of mapped reads by taxonomic group.

## 1.3 Genome size estimations and genome profiling

We conducted genome profiling (estimation of major genome characteristics such as size, heterozygosity, and repetitiveness) on the filtered short-read sequence data with GenomeScope 2.0 (Ranallo-Benavidez et al., 2020; Vurture et al., 2017). Before running GenomeScope 2.0, we counted k-mers with JELLYFISH v2.2.10 (Marçais & Kingsford, 2011) using jellyfish count -C -s 25556999998 -F 3 and a k-mer length of 21 (-m 21) as recommended for most genomes by the authors of GenomeScope2. A histogram of k-mer frequencies was produced with jellyfish histo. GenomeScope 2.0 was run with the exported k-mer count histogram within the online web tool (http://qb.cshl.edu/genomescope/genomescope2.0/) using the following parameters: Kmer length = 21, Read length = 150, Max kmer coverage = 10000. For *H. japonica* we ran the command-line version of Genomescope2 with the option -l = 58 to set the location of the first peak after correspondence with the Genomescope2 developer.


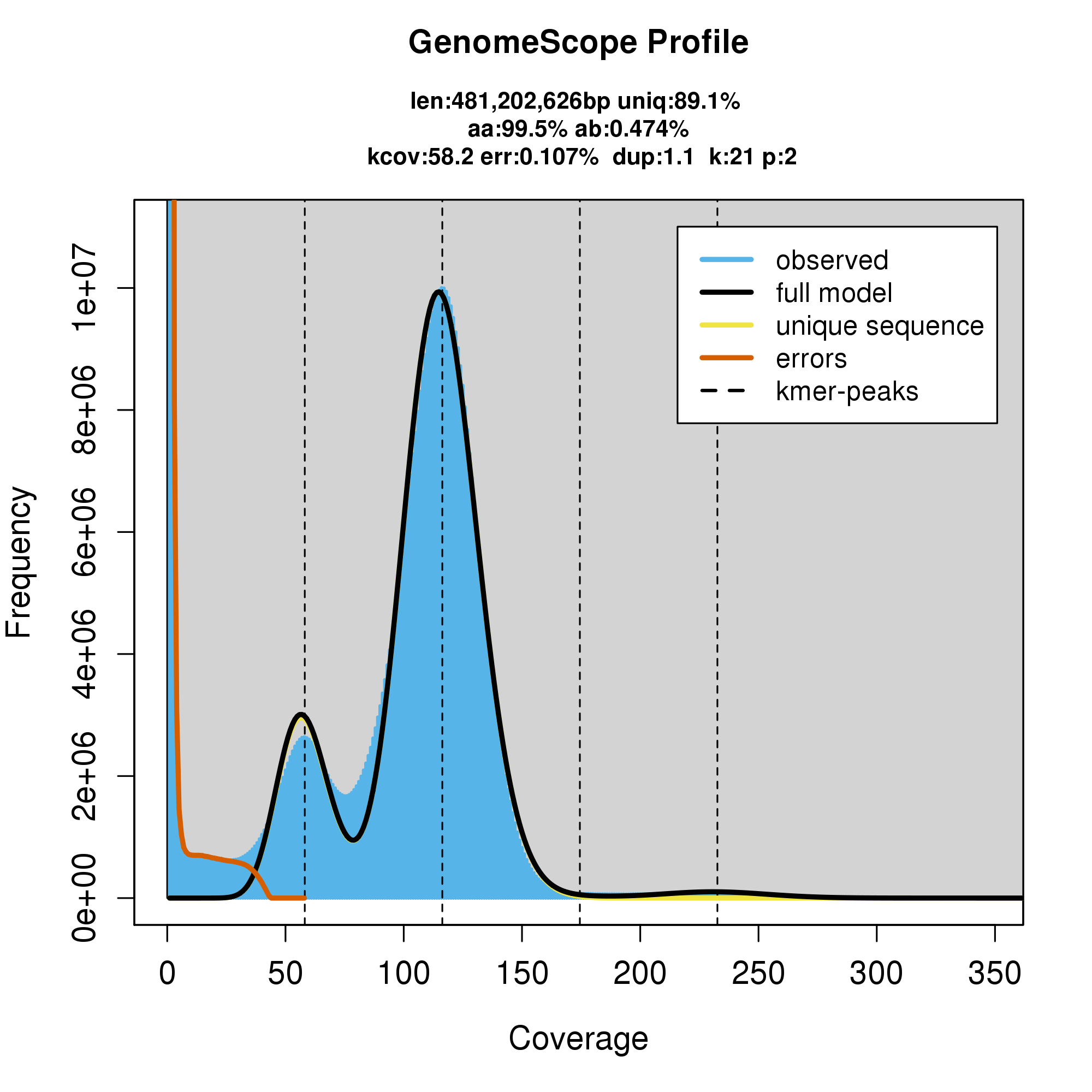

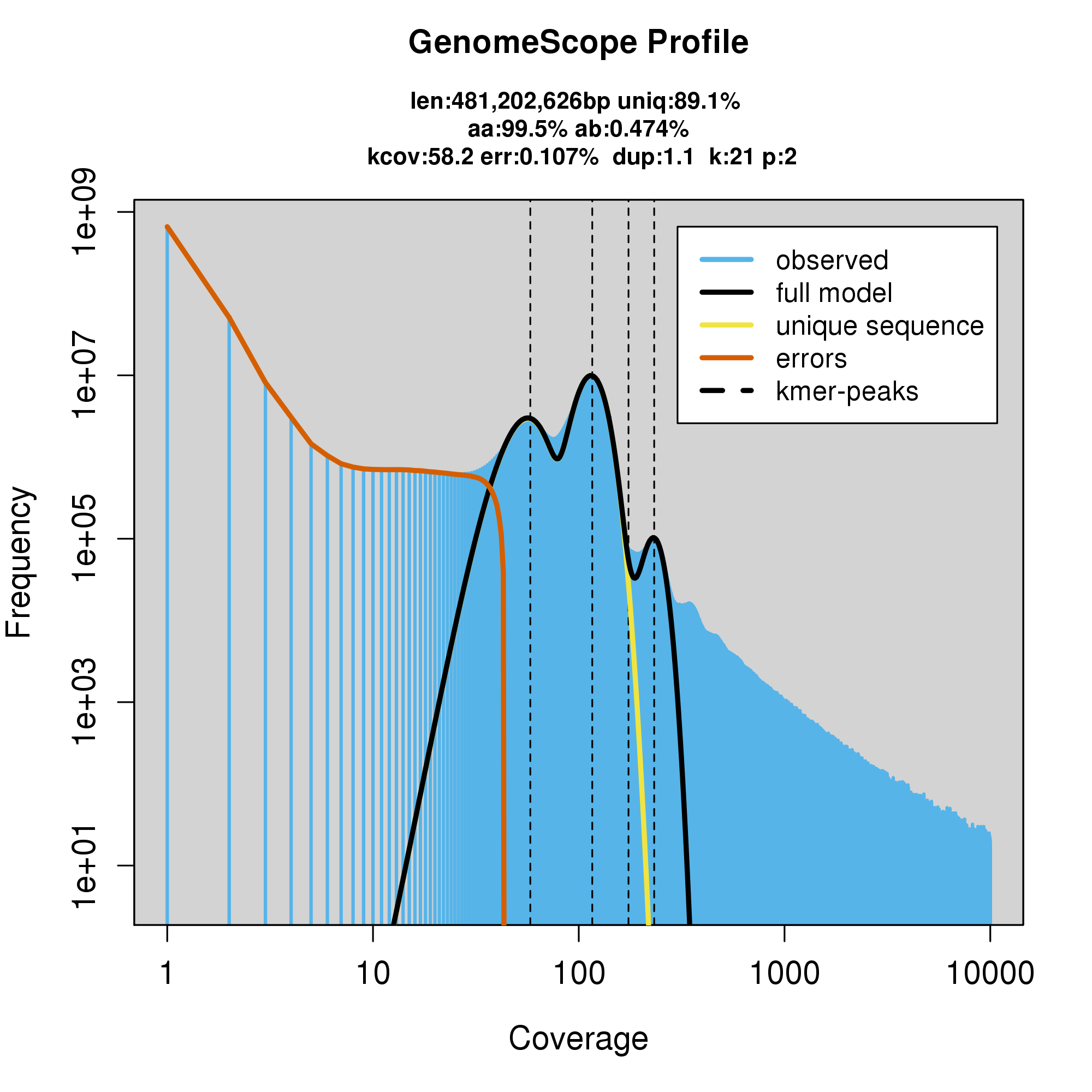


**Supplementary Figure 4: Genomescope2 profile for *H. japonica.*** right: in log scale, len: inferred total genome length, uniq: percent of the genome that is unique, het: overall rate of heterozygosity, kcov: mean kmer coverage for heterozygous bases, err: error rate of the reads, dup: average rate of read duplications. Link to GenomeScope 2 profile: <http://qb.cshl.edu/genomescope/genomescope2.0/analysis.php?code=raff8EDYNv9KgAUHTelt>


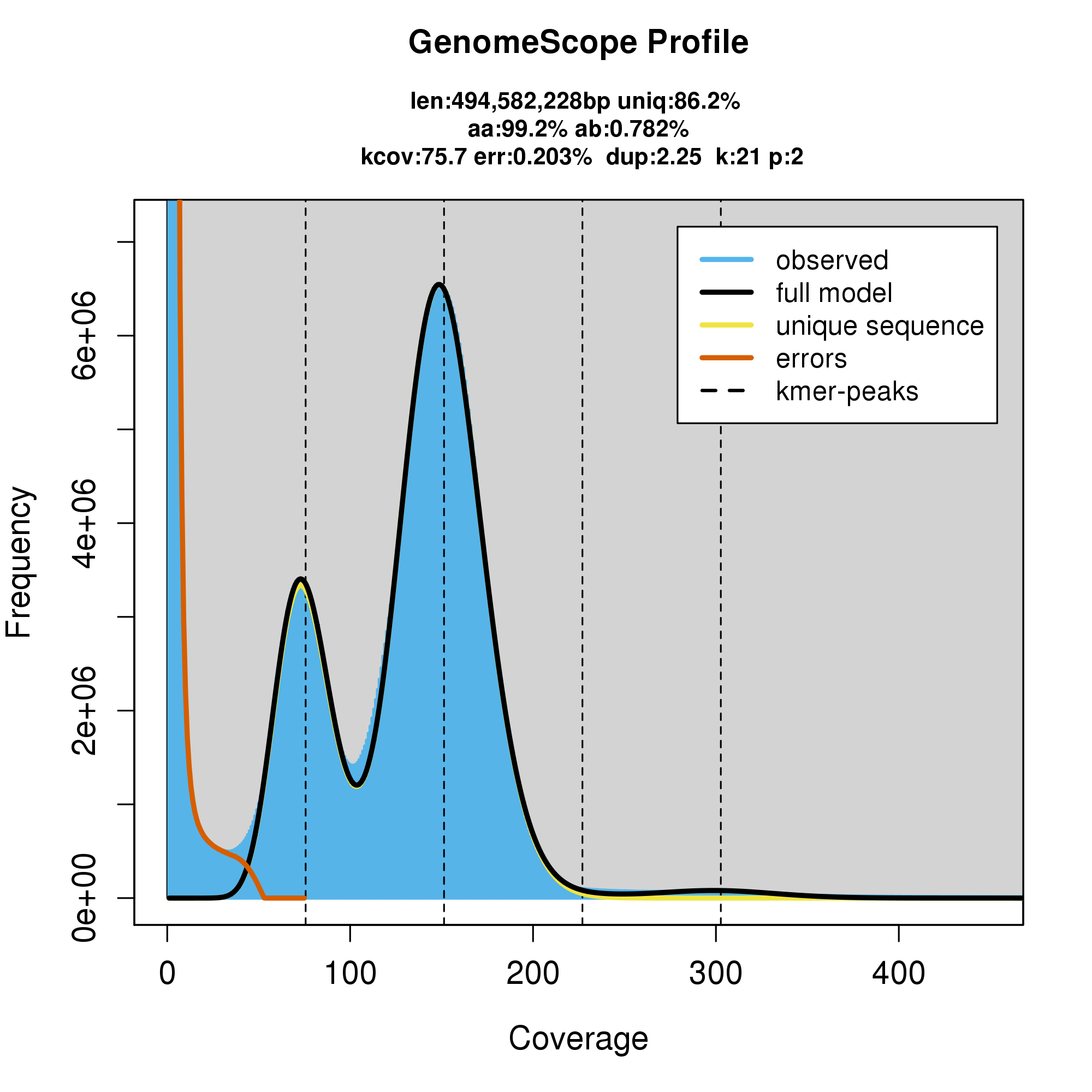

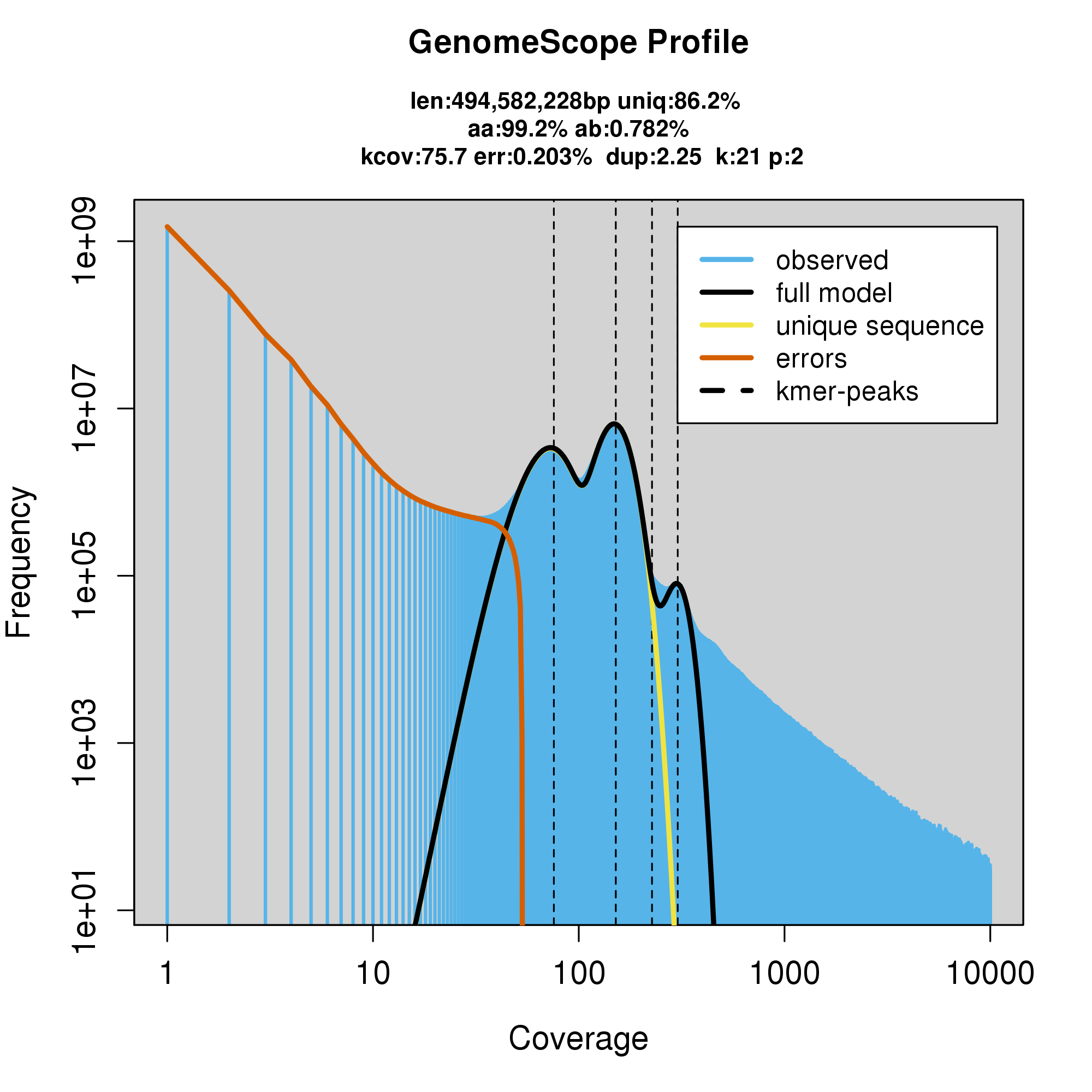


**Supplementary Figure 5: Genomescope2 profile for *H. sp.* (*kuldschensis* group).** right: in log scale, len: inferred total genome length, uniq: percent of the genome that is unique, het: overall rate of heterozygosity, kcov: mean kmer coverage for heterozygous bases, err: error rate of the reads, dup: average rate of read duplications. Link to GenomeScope 2 profile: <http://qb.cshl.edu/genomescope/genomescope2.0/analysis.php?code=ZN2i0yHrxuK4FEWXCxGD>


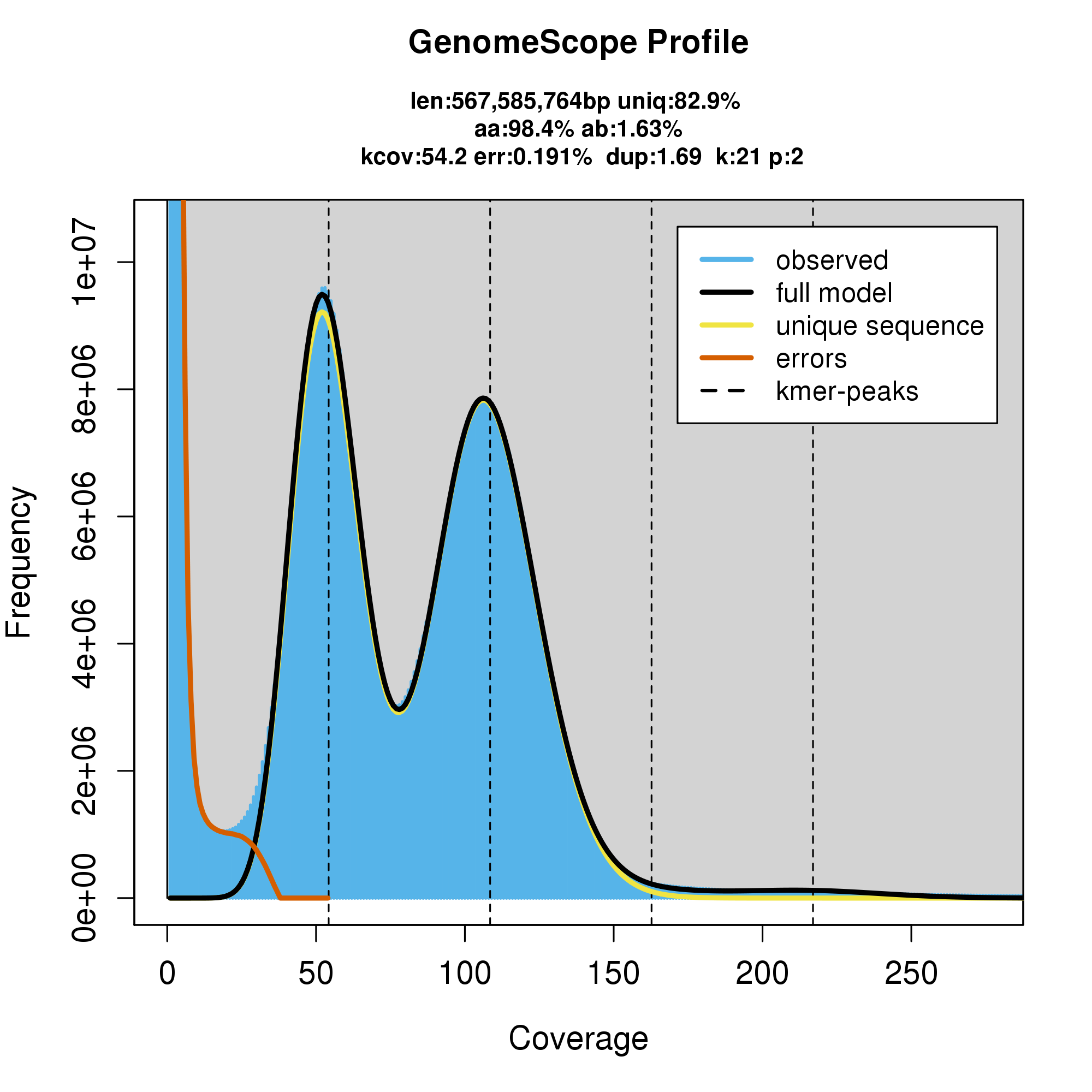

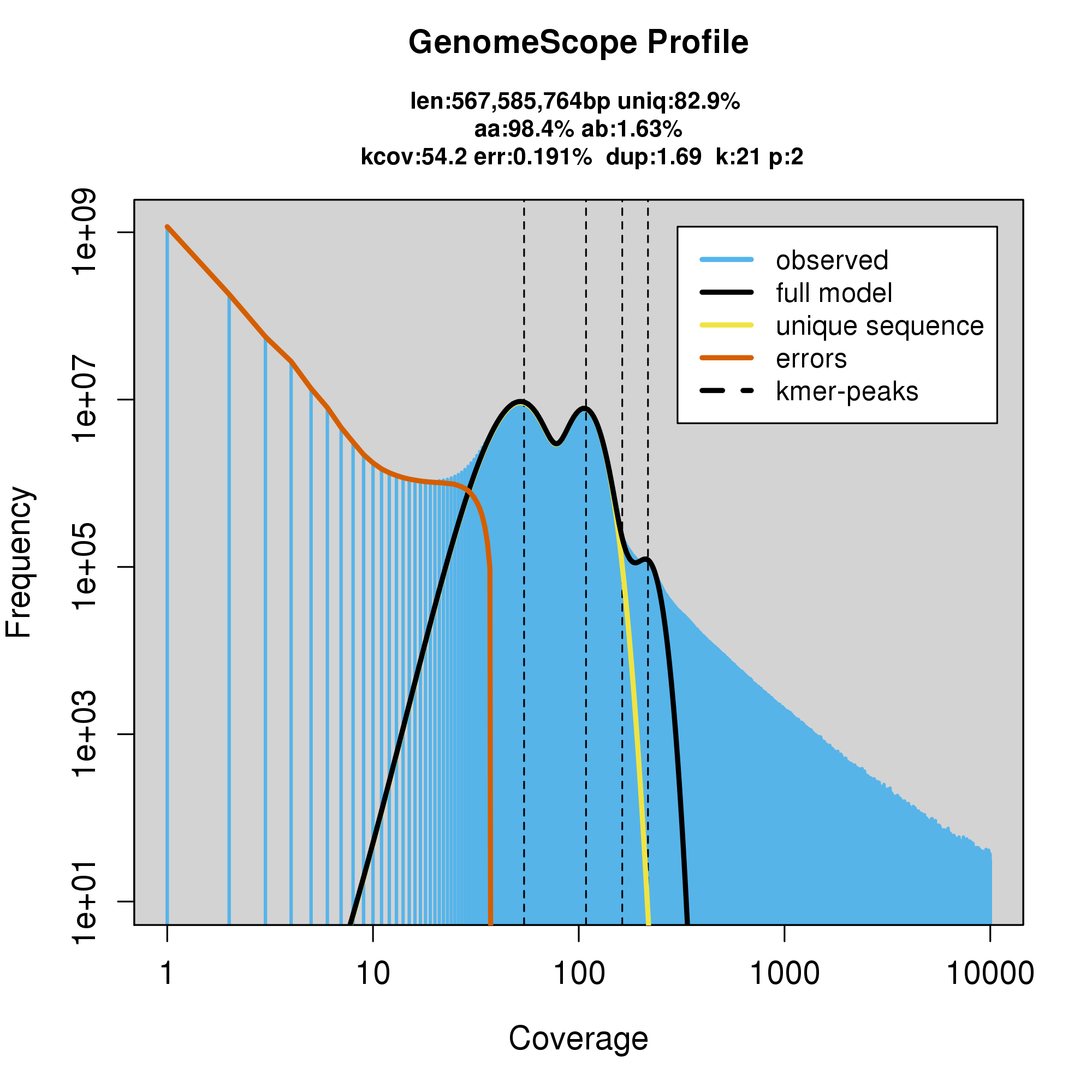


**Supplementary Figure 6: Genomescope2 profile for *H. tibetana.*** right: in log scale, len: inferred total genome length, uniq: percent of the genome that is unique, het: overall rate of heterozygosity, kcov: mean kmer coverage for heterozygous bases, err: error rate of the reads, dup: average rate of read duplications. Link to GenomeScope 2 profile: <http://qb.cshl.edu/genomescope/genomescope2.0/analysis.php?code=DkAhSehXmSYCHCRYMN9n>

## 1.4 *De novo* genomes of three reference species - Repeat annotation

Between 31% (*H. japonica*) and 41-44% (*H. sp.* (*kuldschensis* group) and *H. tibetana*) of the genome assembly was masked as repeats. A high percentage of the repeats were classified as interspersed repeats (approx. 28.5-40.3%). More than half of the interspersed repeats remain unclassified and therefore may be specific to Trichoptera.

**Table S1: Repeat content of *H. japonica***

***
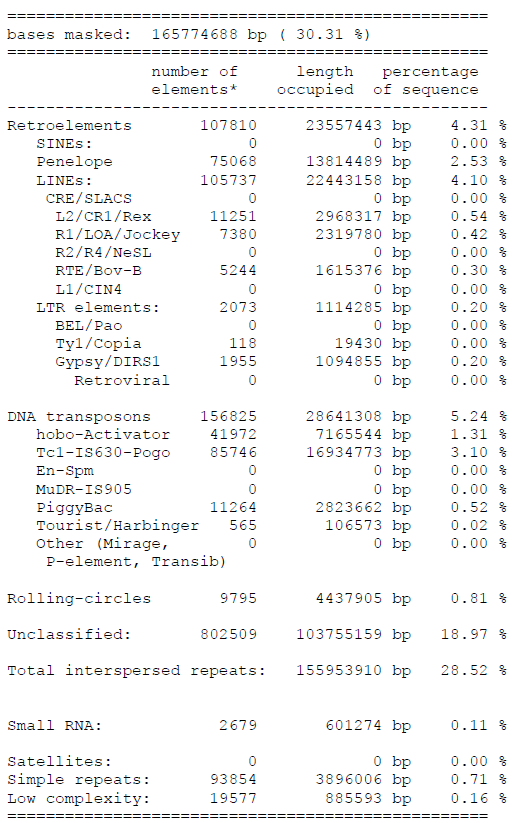
***

**Table S2:** **Repeat content of *H. sp.* (*kuldschensis* group)**

***
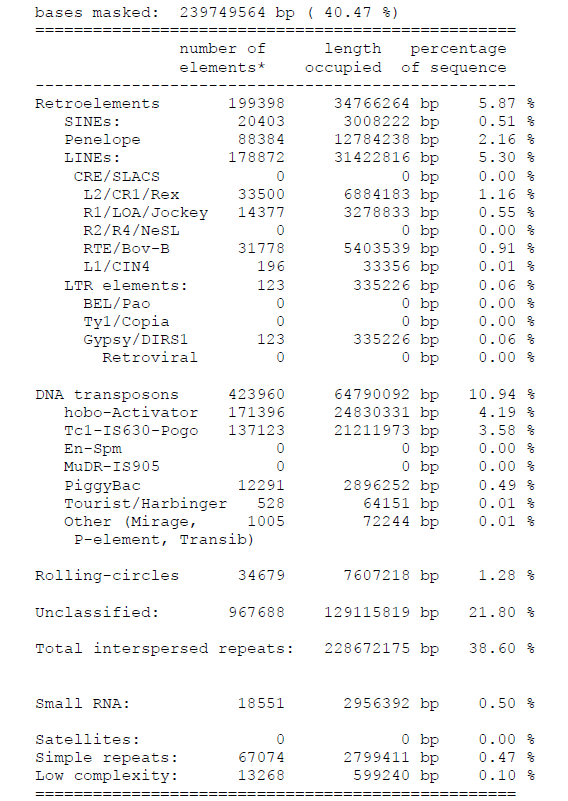
***

**Table S3: Repeat content of *H.tibetana***

***
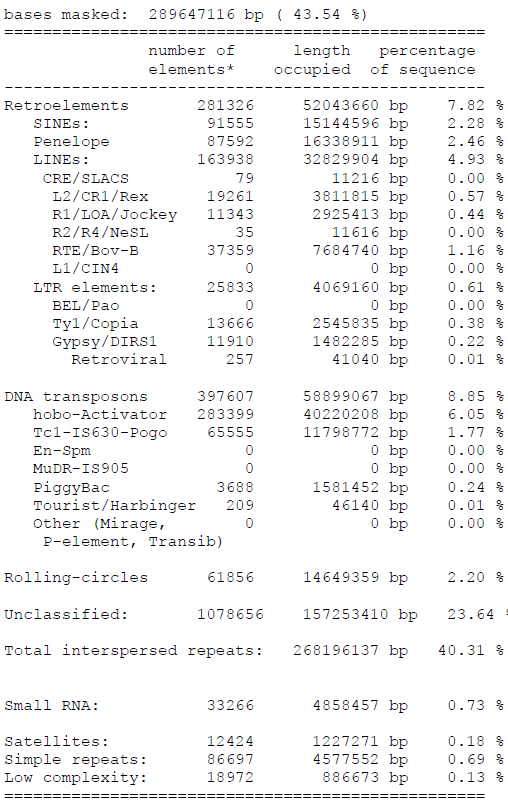
***

## 1.5 Functional annotation of protein coding genes

The annotation of the genomes resulted in the prediction of 9,983 (*H. japonica*), 10,049 (*H. sp.* (*kuldschensis* group)) and 10,994 (*H. tibetana*) proteins. Most of the annotated proteins had functional Blast2GO annotations, were verified by BLAST or were mapped to GO terms. GO Distributions were similar to previously annotated caddisfly genomes. Specifically, the major biological processes were cellular and metabolic processes. Catalytic activity was the largest subcategory in molecular function. Regarding the cellular component category, most genes were assigned to the cell subcategory or to the membrane subcategory.


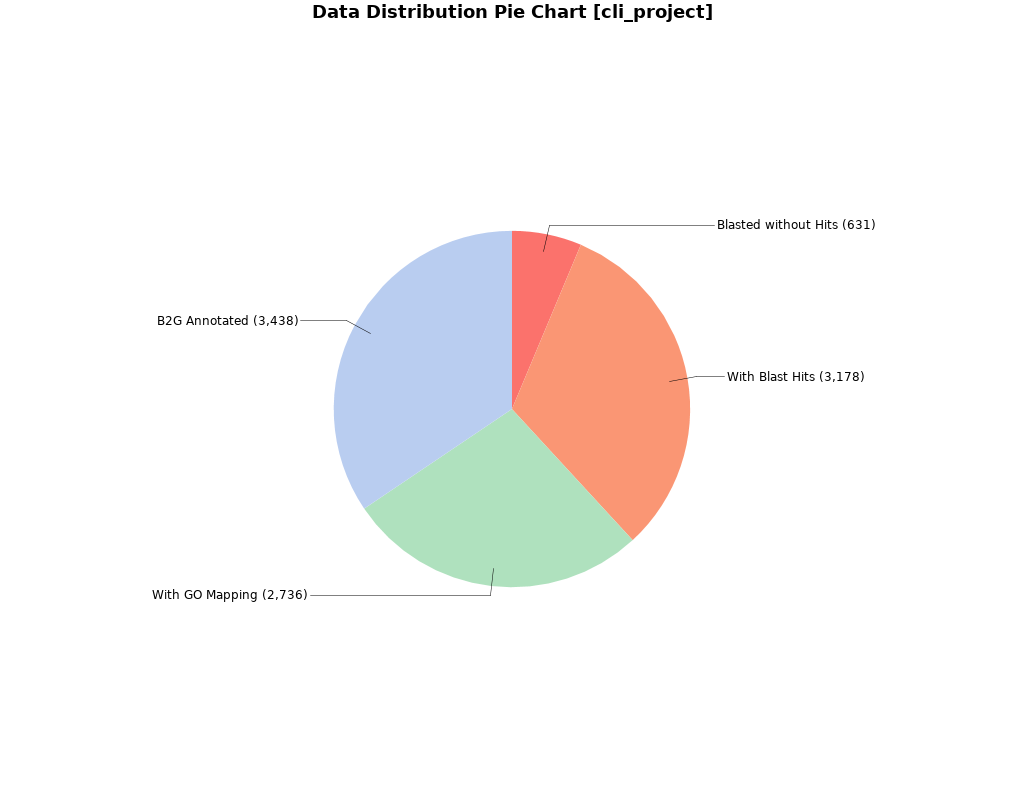


**Supplementary Figure 7:** Blast2Go Annotation Results of ***H. japonica.*** Pie charts showing the percentage of proteins with functional Blast2Go annotations, verified by BLAST and mapped to GO terms.


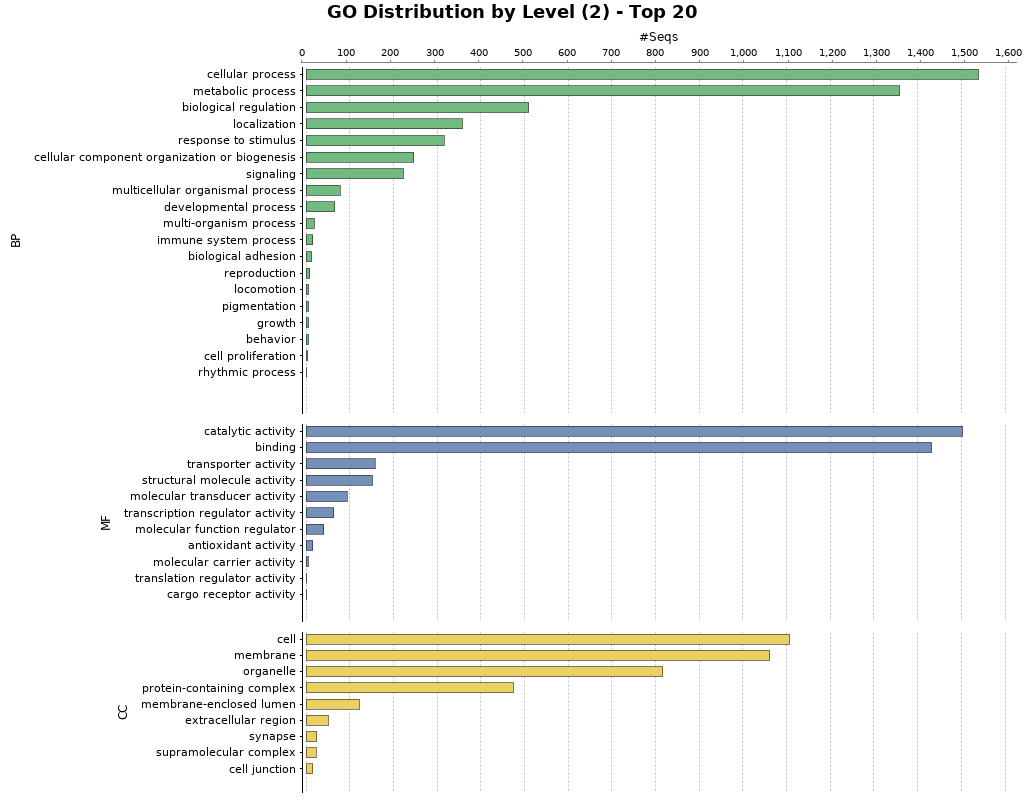


**Supplementary Figure 8: Blast2Go Functional Annotation for *H. japonica*.** Barplot showing GO terms characterized by biological process, molecular function, and cellular component. Barplots are grouped by biological process (BP), molecular function (MF), and cellular component (CC).


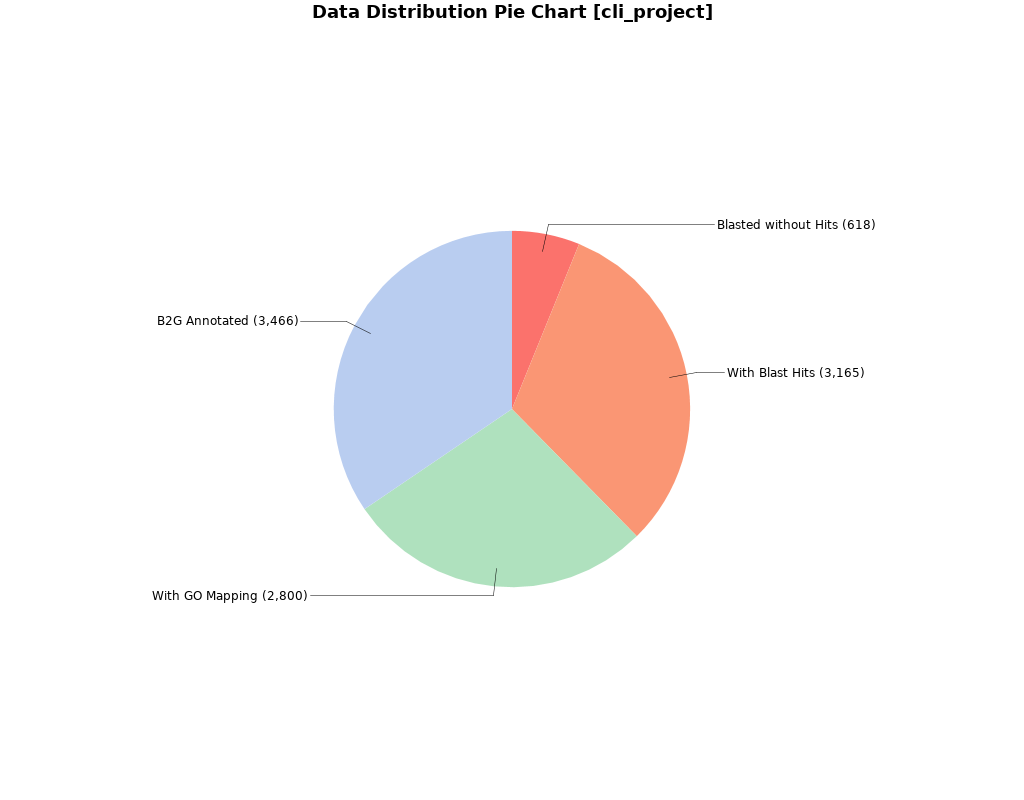


**Supplementary Figure 9: Blast2Go Annotation Results of *H. sp.* (*kuldschensis* group)*.*** Pie charts showing the percentage of proteins with functional Blast2Go annotations, verified by BLAST and mapped to GO terms.


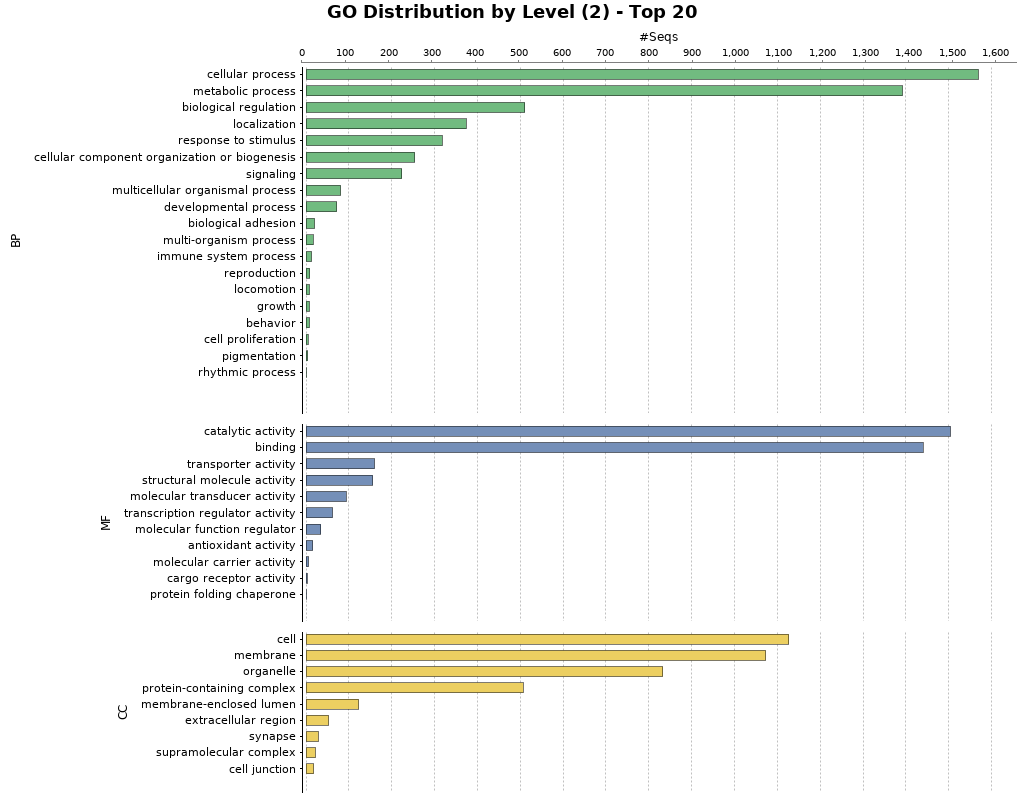


**Supplementary Figure 10: Blast2Go Functional Annotation for *H. sp.* (*kuldschensis* group).** Barplot showing GO terms characterized by biological process, molecular function, and cellular component. Barplots are grouped by biological process (BP), molecular function (MF), and cellular component (CC).


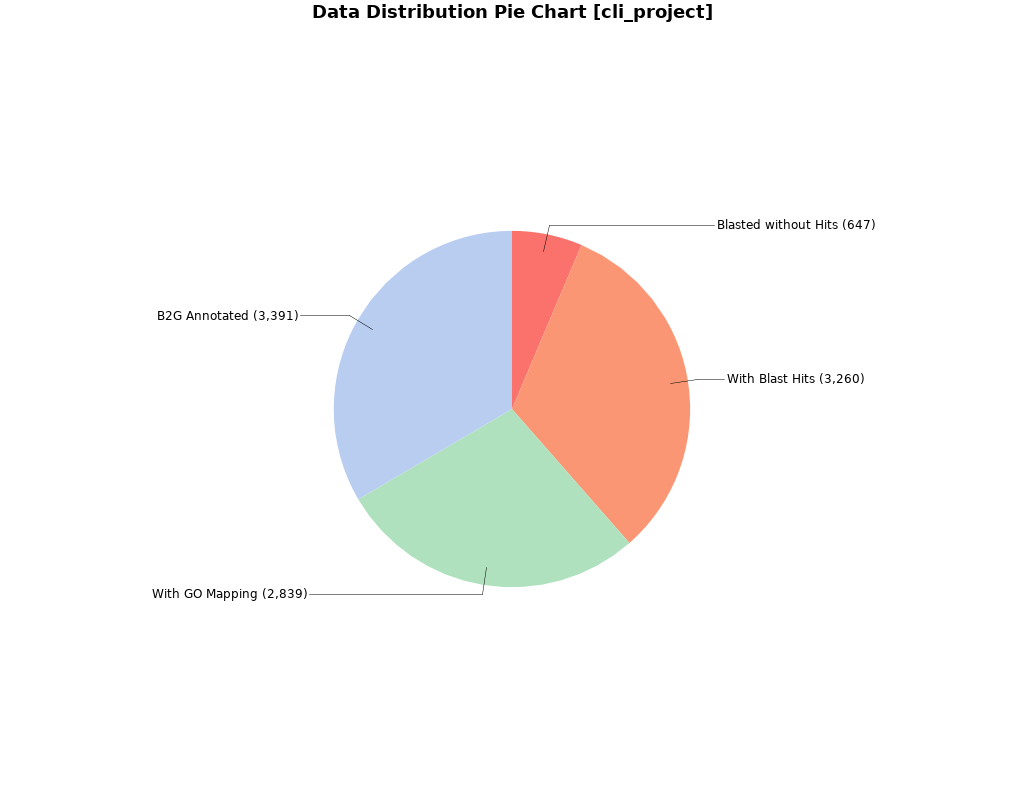


**Supplementary Figure 11: Blast2Go Annotation Results of *H. tibetana*.** Pie charts showing the percentage of proteins with functional Blast2Go annotations, verified by BLAST and mapped to GO terms.

**Supplementary Figure 12: Blast2Go Functional Annotation for *H. tibetana*.** Barplot showing GO terms characterized by biological process, molecular function, and cellular component. Barplots are grouped by biological process (BP), molecular function (MF), and cellular component (CC).
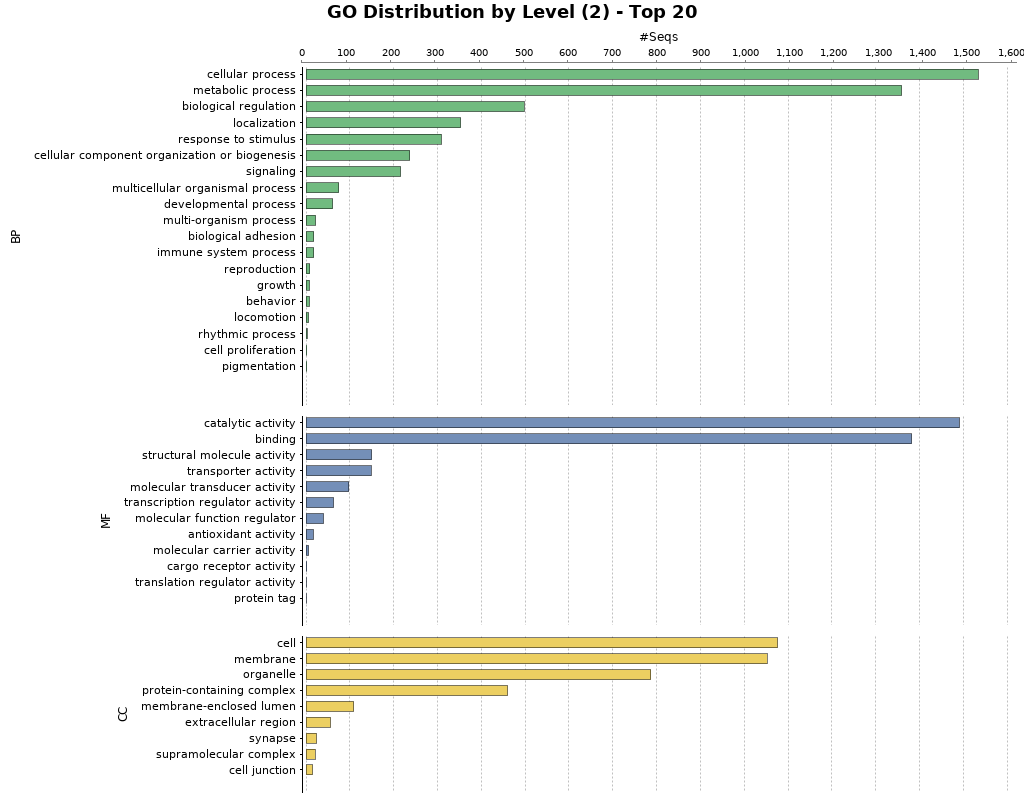


All BUSCO genes, annotation gffs and predicted proteins resulting from GEMOMA, blastp and BLAST2GO results, as well as repeatmodeler and -masker results are available at: https://doi.org/10.6084/m9.figshare.c.6033011.v1.

# 2 Population data processing

## 2.1 Genomic DNA purification

After extracting the genomic DNA, we purified some DNA samples which showed a low DNA quality by using magnetic beads with the following steps: (1) for ~34μL DNA solute, add 70 μL beat suspension (homebrew bead suspension made from SpeedBeads™ magnetic carboxylate modified particles (GE Healthcare) following Rohland & Reich (2012) and mix for 20 minutes on shaker; (2) place it on magnetic rack for 2 minutes, then carefully remove all the supernatant; (3) add 300 μL 70% ethanol; (4) wait 30 seconds, then remove the ethanol; (5) repeat (3) and (4); (6) place back on the magnetic rack; (7) remove the supernatant completely; (8) air dry for 5 minutes; (9) add 30 μL 1× TE buffer (consists of 10 mM Tris-HCL and 1 mM EDTA) and then mix, then incubate on shaker for 15 minutes; (10) pellet the beats on the magnetic rack; (11) transfer the DNA to a new low-bind tube; (12) check the DNA quality. If the quality of the DNA is still low, we also add more purification steps as following: (13) add 3 μL 0.3 M Sodium acetate (NaAc) and mix; (14) then add 75 μL 100% ethanol and mix by inverte; (15) 15 minutes centrifuge at 4 °C and 10000 rpm; (16) remove the supernatant; (17) add 500 μL 80 % ethanol for 10 minutes in the centrifuge (4 °C, 10000 rpm); (18) remove the supernatant; (19) repeat step (17) and (18); (20) air dry for 20 minutes; (21) add 30 μL TE buffer and then check the DNA quality again.

## 2.2 Raw data processing

After quality check and k-mers trimming, Illumina adapter were removed with a custom adapter file (ILLUMINACLIP: all_adapters.fa:2:30:10, SLIDINGWINDOW:4:20 and MINLEN:50) and further processed with Cutadapt v2.23 (Martin, 2011) using the following parameters: -- pair-filter=any -l=140, --max-n=0.

## 2.3 Calling variants with GATK

After the mapping step by Bowtie2, we used SAMtools v1.10 (Danecek et al., 2021) to convert the output sam files to bam format file using option *view -b* and sorted them using option *samtools sort*.

Picard v2.20.8 was used to mark duplicate reads for each dataset with the following steps: 1) create a fasta index on the genome assemblies using SAMtools *faidx* option; 2) create a job file to create a sequence dictionary using *CreateSequenceDictionary* in Picard; 3) mark duplicates using *MarkDuplicates* in Picard, set the maximum number of file handles to keep open when spilling read ends to disk as 1000 by adding the parameter *MAX_FILE_HANDLES_FOR_READ_ENDS_MAP=1000*, as well as the memory for sorting collections as 0.25 by adding the parameter SORTING_COLLECTION_SIZE_RATIO=0.25; 4) index the marked bam files using *index* option in SAMtools.

Afterwards, the genotype calling was conducted in two ways, one was hard calling by VCFtools, the other one was genotype likelihood estimating by Angsd. The hard calling was following these steps: 1) call the SNPs and indels based on local assembly of haplotypes on each of the bam file individually using *HaplotypeCaller* with GATK with options -T HaplotypeCaller -ERC GVCF -pcrModel NONE. We set the threads for native pairHMM implementation as 24 by setting *--native-pair-hmm-threads 24*, and mode for emitting reference confidence scores as *GVCF*; 2) combined the vcf files with same species, same sequencing depth and same reference genome (RG) into one by *CombineGVCFs* option in GATK; 3) call genotype on the combined vcf files using *GenotypeGVCFs* in GATK; 4) select and filter variants by VCFtools. We selected only SNPs and conducted variant- and genotype-level filtering using *--remove-indels*, then all sites with a filter flag were removed by *--remove-filtered-all* option, the SNPs that are not missing in all individuals were kept by setting *--max-missing* as 1. The final outputs were used for estimating the population genetic index.

## 2.4 Generating genotype likelihoods with Angsd

The genotype likelihood was estimated by Angsd using the following settings: SAMtools model was used by setting *-GL* as 1, the reads which were not primary, failure and duplicate, as well as had multiple best hits were removed with *-remove_bads 1* and *-uniqueOnly 1*. The paired reads which mapped both correctly were kept with *-only_proper_pairs 1*. A strict filter on mapping which allows 0 mismatches was conducted by *-C 50*, which means that the mapping quality (MAPQ) score >= 50 were kept. A base alignment quality (BAQ) was performed by *-baq 1*. To ensure the base quality, the bases with a qscore below 30 were discarded with *-minQ 30* and *-minMapQ 30*. The sites contain more than ~ 80% individuals were selected with *-minInd 18* for *H. digitata* samples and *-minInd 19* for *H. tibetana* samples. We also performed an estimation on polymorphic sites by using *-doSnpStat 1* and keep the high confidence polymorphic sites by using *-snp_pval 1e-6*, as well as on divination from Hardy Weinberg equilibrium for each site by using *-doHWE 1* and *-hwe_pval 1e-6*. To estimate the allele frequencies from the genotype likelihoods, we use *-doMajorMinor 1*, *-doMaf 2*, *-doPost 1*, *-minMaf 0.05* settings, and we skip the Triallelic using *-skipTriallelic 1*. For genotype calling, we used *-doGeno 8* settings which will write the posterior probability of all possible genotypes and use *-doGlf 2* which use beagle genotype likelihood format as output. In brief, the command line is:

*angsd -b bamlist.txt -ref $REFERENCE_GENOME -GL 1 -out $OUTPUT -remove_bads 1 -uniqueOnly 1 -only_proper_pairs 1 -C 50 -baq 1 -minMapQ 30 -minQ 30 -minInd 19 -doSnpStat 1 -doHWE 1 -sb_pval 1e-6 -hwe_pval 1e-6 -hetbias_pval 1e-6 -doMajorMinor 1 -skipTriallelic 1 -doMaf 2 -doPost 1 -minMaf 0.05 -snp_pval 1e-6 -doGeno 8 -doGlf 2*

Afterwards, we created a file containing the SNP position based on the output MAF file and counted them. Then we calculated linkage disequilibrium for SNP pairs up to 500 k base pair apart by ngsLD (Fox et al., 2019), and we sampled 5% of the SNP and plotted it as a LD decay model by fit_LDdecay.R in ngsLD. Next, we pruned the linked sites using prune_graph.pl with these parameters: maximum distance between nodes was setted as 50 kb by *--max_kb_dist 50*, minimum weight of an edge to assume nodes are connected was setting as 0.1 by *--min_weight 0.1*. In the end, we extracted the pruned sites and used it for PCA and admixture estimation.

We use VCFtools for estimating the population differentiation of the four population of *H. digitata* and *H. tibetana*, including nucleotide diversity (π value), individual heterozygosity, and *F*_ST_. The nucleotide diversity and *F*_ST_ were calculated with a 50 kb window size by the parameters *--fst-window-size 50000* and *--window-pi 50000*. We employed PCAngsd for PCA and [NGSadmix](http://www.popgen.dk/software/index.php/NgsAdmix) for admixture proportions estimating.

# 3 Plotting

*### Fig. 4*

*###++++++++++++++ plot in r (genome wide FST) ++++++++++++++++++++*

*# plot genome wide FST of all the data in a loop*

*files <- list.files()*

*#digitata*

*for(i in 1:length(files)) {*

*df <- read.table(paste0(files[i]), header = TRUE)*

*df[df < 0] <- 0*

*plot <- ggplot(df) +*

*geom_point(aes(x = Rank, y = WEIGHTED_FST, colour= WEIGHTED_FST > 0.16)) +*

*scale_colour_manual(name = 'PC2 > 0.16', values = setNames(c('#005AB5','#999999'),c(T, F))) + theme_classic()+*

*geom_hline(yintercept = 0.16, colour = "#999999") +*

*theme(legend.position="none") +*

*ylim(0.0, 1.0) + xlim(0.0, 13050) +*

*xlab(NULL) + labs(y="FST")*

*pdf(paste0(file = files[i],".pdf"), width = 8, height = 4)*

*print(plot)*

*dev.off()*

*}*

*#tibetana*

*for(i in 1:length(files)) {*

*df <- read.table(paste0(files[i]), header = TRUE)*

*df$Rank <- 1:nrow(df) #add a column with consecutive numbers*

*df[df < 0] <- 0*

*df$WEIGHTED_FST <- as.numeric(df$WEIGHTED_FST)*

*plot <- ggplot(df) +*

*geom_point(aes(x = Rank, y = WEIGHTED_FST, colour= WEIGHTED_FST > 0.46)) +*

*scale_colour_manual(name = 'PC2 > 0.46', values = setNames(c('#DC3220','#999999'),c(T, F))) + theme_classic()+*

*geom_hline(yintercept = 0.46, colour = "#999999") +*

*theme(legend.position="none") +*

*ylim(0.0, 1.0) + xlim(0.0, 13899) +*

*xlab(NULL) + labs(y="FST")*

*pdf(paste0(file = files[i],".pdf"), width = 8, height = 4)*

*print(plot)*

*dev.off()*

*}*

*### Fig. 5*

*###++++++++++++++ plot in r (PCA) ++++++++++++++++++++*

*library(ggplot2)*

*library(plot3D)*

*library(admixturegraph)*

*library(gridExtra)*

*library( ggpubr)*

*library(dplyr)*

*#Prepare the data and perform the PCA.*

*# set working directory*

*setwd($DIR)*

*# read bamlist used with ANGSD*

*samples <- read.table("POPinfo.txt")[,2]*

*samples <- as.factor(samples)*

*# set subspecies*

*pop <- read.table("POPinfo.txt")[,1]*

*pop <- as.factor(pop)*

*files <- list.files(pattern = ".cov")*

*for(i in 1:length(files)) {*

*# read covariance matrix generated with PCAngsd*

*tibetana_cov <- as.matrix(read.table(paste0(files[i])))*

*# append sample names as row and column names to covariance matrix*

*dimnames(tibetana_cov) <- list(samples, samples)*

*# perform PCA*

*pca <- prcomp(tibetana_cov, scale = TRUE)*

*summary(pca)*

*###Plot the eigenvalues of the principal components.*

*# get the eigenvalues as percentage*

*eigenval <- pca$sdev^2*

*explained_var <- 100*(eigenval/sum(eigenval))*

*# create a data frame of explained variance per principal component*

*df <- data.frame(prin_comp = c(seq(1, length(eigenval))), explained_var)*

*# generate the plot*

*p <- ggplot(df, aes(prin_comp, explained_var)) +*

*# color principal components 1-3 in steelblue and the remaining in grey40*

*geom_col(fill = c(rep("steelblue", 3), rep("grey40", length(eigenval)-3))) +*

*# change x label*

*xlab("Principal components") +*

*# change y label*

*ylab("Explained variance") +*

*# change title and axis font size*

*theme(axis.title = element_text(size = 14),*

*axis.text = element_text(size = 11))*

*###savme plot*

*pdf(paste0(file = files[i],"_explained_variance.pdf"), width = 8, height = 4)*

*print(p)*

*dev.off()*

*### plot pca*

*pdf(paste0(file = files[i],"_2d.pdf"), width = 5, height = 4)*

*# set plot layout*

*layout(matrix(c(1, 2, 3, 3), ncol = 2, byrow = TRUE), heights = c(5, 0.7))*

*# plot PC1 and PC2*

*plot(pca$x[, c(1, 2)],pch = 16,*

*col = c("skyblue2", "hotpink2", "darkseagreen3", "burlywood2")[pop], mgp = c(2.3, 1, 0), cex = 1.2, cex.lab = 1.3,*

*xlab = paste("PC1 (", round(explained_var[1], 2), "%)", sep = ""),*

*ylab = paste("PC2 (", round(explained_var[2], 2), "%)", sep = ""))*

*abline(v = 0, h = 0, col = "dark grey", lty = 2)*

*# plot PC1 and PC3*

*plot(pca$x[, c(1, 3)], pch = 16,*

*col = c("skyblue2", "hotpink2", "darkseagreen3", "burlywood2")[pop], mgp = c(2.3, 1, 0), cex = 1.2, cex.lab = 1.3,*

*xlab = paste("PC1 (", round(explained_var[1], 2), "%)", sep = ""),*

*ylab = paste("PC3 (", round(explained_var[3], 2), "%)", sep = ""))*

*abline(v = 0, h = 0, col = "dark grey", lty = 2)*

*# set margins*

*par(mar = c(0, 0, 0, 0))*

*# start new frame*

*plot.new()*

*# plot legend*

*legend(x = "bottom", ncol = 4,legend = c("pop1","pop2","pop3","pop4"), pch = 16,*

*col = c("skyblue2", "hotpink2", "darkseagreen3", "burlywood2"),*

*cex = 1.2, title = "population", box.lty = 0)*

*dev.off()*

*### 3D plot*

*plot.new()*

*pdf(paste0(file = files[i],"_3d.pdf"), width = 7, height = 7)*

*# set the plot layout*

*layout(matrix(c(1, 1, 1, 0,*

*1, 1, 1, 0,*

*1, 1, 1, 2,*

*1, 1, 1, 2),*

*nrow = 4, ncol = 4, byrow = TRUE))*

*# set margins*

*par(mar = c(0, 0, 0, 0))*

*# plot PC1, PC2, and PC3*

*scatter3D(pca$x[, 1], pca$x[, 2], pca$x[, 3],*

*colvar = NULL, colkey = FALSE, pch = 16, col = c("skyblue2", "hotpink2", "darkseagreen3", "burlywood2")[pop],*

*main = NULL, cex.main=1.5,*

*groups = pop, surface = FALSE, grid = FALSE, ellipsoid = TRUE,*

*cex = 3, cex.lab = 2, bty = "b2", theta = 30, phi = 45,*

*xlab = paste("PC1 (", round(explained_var[1], 2), "%)", sep = ""),*

*ylab = paste("PC2 (", round(explained_var[2], 2), "%)", sep = ""),*

*zlab = paste("PC3 (", round(explained_var[3], 2), "%)", sep = ""))*

*dev.off()*

*}*

*###++++++++++++++ plot in r (admixture) ++++++++++++++++++++*

*### Make a boxplot of run likelihoods per K.*

*# load package*

*library(ggplot2)*

*library(gridExtra)*

*library( ggpubr)*

*library(dplyr)*

*setwd("/MY_DIR/$DATA")# setwd to each subdirectories*

*# read likelihoods list*

*fin <- read.table("likelihoods.list")*

*# add column with value of K*

*data <- cbind(c(rep("2", 10), rep("3", 10), rep("4", 10)), fin)*

*# add headers*

*colnames(data) <- c("K", "Likelihoods")*

*# convert the variable K to a factor variable*

*data$K <- as.factor(data$K)*

*# create basic plot*

*pdf("Likelihoods.pdf", width = 6, height = 4)*

*ggplot(data, aes(x = K, y = Likelihoods)) +*

*# add boxplot*

*geom_boxplot(outlier.shape = NA) +*

*# add stripchart*

*geom_jitter(aes(colour = "red", alpha = 0.5), size = 2, width = 0.2) +*

*# change x label*

*xlab("K") +*

*# change title and axis font size and remove legend*

*theme(axis.title = element_text(size = 14),*

*axis.text = element_text(size = 11),*

*legend.position = "none")*

*dev.off()*

*### plot population structure with sample name*

*# read population labels and estimated admixture proportions*

*pop<-read.table("POPinfo.txt",as.is=T)*

*q2<-read.table("$SPECIES_.k2.qopt")*

*q3<-read.table("$SPECIES_.k3.qopt")*

*q4<-read.table("$SPECIES_.k4.qopt")*

*# plot*

*pdf("ngsadmix.pdf", width = 7, height = 7)*

*# order according to population*

*ord<-order(pop[,1])*

*par(mfrow=c(3,1))*

*p2 <-barplot(t(q2)[,ord],col=2:5,space=0,border=NA,xlab="Individuals",ylab="K=2") +*

*text(tapply(1:nrow(pop),pop[ord,1],mean),-0.05,unique(pop[ord,1]),xpd=T) +*

*abline(v=cumsum(sapply(unique(pop[ord,1]),function(x){sum(pop[ord,1]==x)})),col=1,lwd=1.2)*

*p3 <-barplot(t(q3)[,ord],col=2:5,space=0,border=NA,xlab="Individuals",ylab="K=3") +*

*text(tapply(1:nrow(pop),pop[ord,1],mean),-0.05,unique(pop[ord,1]),xpd=T) +*

*abline(v=cumsum(sapply(unique(pop[ord,1]),function(x){sum(pop[ord,1]==x)})),col=1,lwd=1.2)*

*p4 <-barplot(t(q4)[,ord],col=2:5,space=0,border=NA,xlab="Individuals",ylab="K=4") +*

*text(tapply(1:nrow(pop),pop[ord,1],mean),-0.05,unique(pop[ord,1]),xpd=T) +*

*abline(v=cumsum(sapply(unique(pop[ord,1]),function(x){sum(pop[ord,1]==x)})),col=1,lwd=1.2)*

*dev.off()*

*### Fig. 6*

*###++++++++++++++ plot in r (PCA) ++++++++++++++++++++*

*### plot pca with HT&12.5x*

*setwd($DIR)*

*library(ggplot2)*

*library(plot3D)*

*library(admixturegraph)*

*library(gridExtra)*

*library( ggpubr)*

*library(dplyr)*

*# read bamlist used with ANGSD*

*samples <- read.table("$SPECIES_HT12.5_POPinfo.txt")[,1]*

*samples <- as.factor(samples)*

*# set subspecies*

*pop <- read.table("$SPECIES_HT12.5_POPinfo.txt")[,2]*

*pop <- as.factor(pop)*

*# read covariance matrix generated with PCAngsd*

*$SPECIES_cov <- as.matrix(read.table("$SPECIES_Him_HT1_12.5.ld_pruned.beagle.gz.cov"))*

*# append sample names as row and column names to covariance matrix*

*dimnames($SPECIES_cov) <- list(samples, samples)*

*# perform PCA*

*pca <- prcomp($SPECIES_cov, scale = TRUE)*

*summary(pca)*

*###Plot the eigenvalues of the principal components.*

*# get the eigenvalues as percentage*

*eigenval <- pca$sdev^2*

*explained_var <- 100*(eigenval/sum(eigenval))*

*# create a data frame of explained variance per principal component*

*df <- data.frame(prin_comp = c(seq(1, length(eigenval))), explained_var)*

*#pdf("digitata_2d_HT12.5.pdf", width=10, height=8) #digitata*

*pdf("tibetana_2d_HT12.5.pdf", width=10, height=8) #tibetana*

*# set plot layout*

*layout(matrix(c(1, 2, 3, 3), ncol = 2, byrow = TRUE), heights = c(5, 0.7))*

*# plot PC1 and PC2*

*plot(pca$x[, c(1, 2)],pch = 16,*

*#col = c("#022C4F", "#054A9E", "#0789E2", "#80CEF9")[pop], # digitata*

*col = c("#660603", "#BA0C08", "#F95D5D", "#F9A29B")[pop], # tibetana*

*mgp = c(2.3, 1, 0), cex = 2.7, cex.lab = 1.3,*

*xlab = paste("PC1 (", round(explained_var[1], 2), "%)", sep = ""),*

*ylab = paste("PC2 (", round(explained_var[2], 2), "%)", sep = ""))*

*abline(v = 0, h = 0, col = "dark grey", lty = 2)*

*# plot PC1 and PC3*

*plot(pca$x[, c(1, 3)], pch = 16,*

*#col = c("#022C4F", "#054A9E", "#0789E2", "#80CEF9")[pop], # digitata*

*col = c("#660603", "#BA0C08", "#F95D5D", "#F9A29B")[pop], # tibetana*

*mgp = c(2.3, 1, 0), cex = 2.7, cex.lab = 1.3,*

*xlab = paste("PC1 (", round(explained_var[1], 2), "%)", sep = ""),*

*ylab = paste("PC3 (", round(explained_var[3], 2), "%)", sep = ""))*

*abline(v = 0, h = 0, col = "dark grey", lty = 2)*

*# set margins*

*par(mar = c(0, 0, 0, 0))*

*# start new frame*

*plot.new()*

*# plot legend*

*legend(x = "bottom", ncol = 4,legend = c("pop1","pop2","pop3","pop4"), pch = 16,*

*#col = c("#022C4F", "#054A9E", "#0789E2", "#80CEF9"), # digitata*

*col = c("#660603", "#BA0C08", "#F95D5D", "#F9A29B"), # tibetana*

*cex = 1.5, box.lty = 0)*

*# save plot*

*dev.off()*

*###++++++++++++++ plot in r (heterozygosity) ++++++++++++++++++++*

*### bar plot of heterozygosity*

*setwd($DIR)*

*library(ggplot2)*

*library(reshape2)*

*library(grid)*

*#Het_ind <- read.table("Het_digitata_plots.txt", header = T) # digitata populations*

*Het_ind <- read.table("Het_tibetana_plots.txt", header = T)*

*# plot*

*# box plot*

*means <- aggregate(F ~ Pop, Het_ind, mean)*

*Het_plot_box <-ggplot(Het_ind, aes(Pop, F, color=Pop)) +*

*#scale_color_manual(values = c("#022C4F","#054A9E","#0789E2","#80CEF9"), # digitata populations*

*scale_color_manual(values = c("#660603","#BA0C08","#F95D5D","#F9A29B"), # tibetana populations*

*breaks = c("pop1", "pop2","pop3","pop4")) +geom_boxplot() +*

*theme_classic() + ylim(0, 0.4)+*

*theme(text = element_text(size=12),*

*axis.title.x = element_blank(), axis.text.x = element_blank(),*

*axis.title.y = element_text(), axis.text.y = element_text())+*

*stat_summary(fun=mean, colour="#139B7A", geom="point",*

*shape=18, size=3, show.legend=FALSE) +*

*geom_text(data = means, aes(label = round(F, 2), y = F + 0.02), size = 4)*

*Het_plot_box*

*# bar plot*

*Het_plot_bar <- ggplot(Het_ind, aes(Individual, F, fill = Pop)) +*

*#scale_fill_manual(values = c("#022C4F","#054A9E","#0789E2","#80CEF9"), # digitata populations*

*scale_fill_manual(values = c("#660603","#BA0C08","#F95D5D","#F9A29B"), # tibetana populations*

*breaks = c("pop1", "pop2","pop3","pop4")) +*

*geom_col(colour = "black", size = 0.1) +*

*scale_x_discrete(limits=Het_ind$Individual) +*

*theme_classic() + ylim(0, 0.4)+*

*theme(text = element_text(size=12),*

*axis.text.x = element_text(),*

*axis.title.x = element_blank(),*

*axis.title.y = element_text(),*

*axis.text.y = element_text())*

*Het_plot_bar*

*# stack the two plots in one figure*

*# pdf("Het_digitata_HT&12.5.pdf",,*

*pdf("Het_tibetana_HT&12.5.pdf",*

*width = 10,*

*height = 8)*

*grid.newpage()*

*grid.draw(rbind(ggplotGrob(Het_plot_box), ggplotGrob(Het_plot_bar), size = "last"))*

*# Save as pdf*

*dev.off()*

*###++++++++++++++ plot in r (Pi) ++++++++++++++++++++*

*###plot in R*

*setwd($DIR)*

*#library*

*library(ggplot2)*

*library(gridExtra)*

*library( ggpubr)*

*library(dplyr)*

*#load file in R*

*# digitata_pi <- read.table("digitata_pi_extracted.txt", header = TRUE)*

*tibetana_pi <- read.table("tibetana_pi_extracted.txt", header = TRUE)*

*# digitata_pi <- data.frame(digitata_pi)*

*tibetana_pi <- data.frame(tibetana_pi)*

*###---> subset dataset based on RC (reference & depth)*

*###H.tibetana&12.5*

*# df_tibetana12.5 <- digitata_pi %>% filter(RC %in% c("H.tibetana12.5"))*

*df_tibetana12.5 <- tibetana_pi %>% filter(RC %in% c("H.tibetana12.5"))*

*head(df_tibetana12.5)*

*# plot*

*means <- aggregate(Pi ~ Population, df_tibetana12.5, mean)*

*p_tibetana12.5 <-ggplot(df_tibetana12.5, aes(Population, Pi, color=Population)) +*

*# scale_color_manual(values = c("#022C4F","#054A9E","#0789E2","#80CEF9"), #digitata*

*scale_color_manual(values = c("#660603","#BA0C08","#F95D5D","#F9A29B"), #tibetana*

*breaks = c("pop1", "pop2","pop3","pop4")) +geom_boxplot() +*

*#theme_classic() + ylim(0, 0.004)+ # digitata*

*theme_classic() + ylim(0, 0.0063)+ # tibetana*

*theme(text = element_text(size=12),*

*axis.title.x = element_blank(), axis.text.x = element_blank(),*

*axis.title.y = element_text(), axis.text.y = element_text())+*

*scale_fill_discrete(breaks=c("pop1","pop2","pop3","pop4"))+*

*stat_summary(fun=mean, colour="#139B7A", geom="point",*

*shape=18, size=3, show.legend=FALSE) +*

*geom_text(data = means, aes(label = round(Pi, 4), y = F + 0.003), size = 3)*

*p_tibetana12.5*

*# stack the two plots in one figure*

*#pdf("Pi_digitata_HT&12.5.pdf",*

*pdf("Pi_tibetana_HT&12.5.pdf",*

*width = 10,*

*height = 4)*

*p_tibetana12.5*

*# Save as pdf*

*dev.off()*

# 4 Results

After reads of the 30 datasets (2 species × 3 depths × 5 RGs) were mapped to the five RGs separately, variant sites were called using GATK, and genotype likelihoods were estimated using ANGSD. The number of filtered SNPs varied greatly depending on RG, sequencing depth, as well as the target species itself (Fig. 4). The number of SNPs sharply decreased when the genetic relatedness with RG decreased for all datasets. This was particularly the case for the *H. tibetana* populations using ANGSD to call the variants, which dropped from millions to thousands when mapping to the other RGs. Likewise, the number of SNPs decreased drastically when sampling depth decreased from 12.5× to 3.5× for all datasets. In addition, the number of SNPs called by GATK was generally higher in *H. tibetana* populations than in *H. digitata* populations when using the same depth or RG, but the same which was estimated by Angsd except using *H. tibetana* as RG.

To estimate and compare population diversity among the datasets based upon different RGs and contrasting depths, we estimated individual *F*, nucleotide diversity (π) of populations, as well as *F*_ST_ of all pairwise populations using the SNPs estimated by GATK. For the estimates of *F*, the results showed that the *F* value of each individual was inversely correlated with sequencing depth, but consistent among different RGs in most of the cases (Supplementary Figure 14). For example, the *F* value of each individual within both *H. tibetana* and *H. digitata* increased approximately two-fold if we decreased the depth from 12.5× to 3.5× (keep the RG the same, e.g., *H. tibetana*), but was held constant if we only change the RG (e.g., from *H. tibetana* to *R. brunnea*, with the same depth, e.g., 12.5×). However, even though the value of *F* varied with depth, all datasets revealed consistent patterns of homozygosity for both species: *F* values of all individuals were similar. *F* values showed greater fluctuation when mapped to an RG that is distantly related to the target species and with low depth. For instance, the *F* values among individuals of *H. digitata* were irregular when using *H. japonica* as RG and 3.5× depth; for *H. tibetana*, *F* was unstable when mapped to *H. sp.* (*kuldschensis* group). In summary, for individual *F* estimates, a distantly related RG or lower sequence depth may cause numerical bias, but they remain sufficient to reflect a general homozygosity pattern among populations.


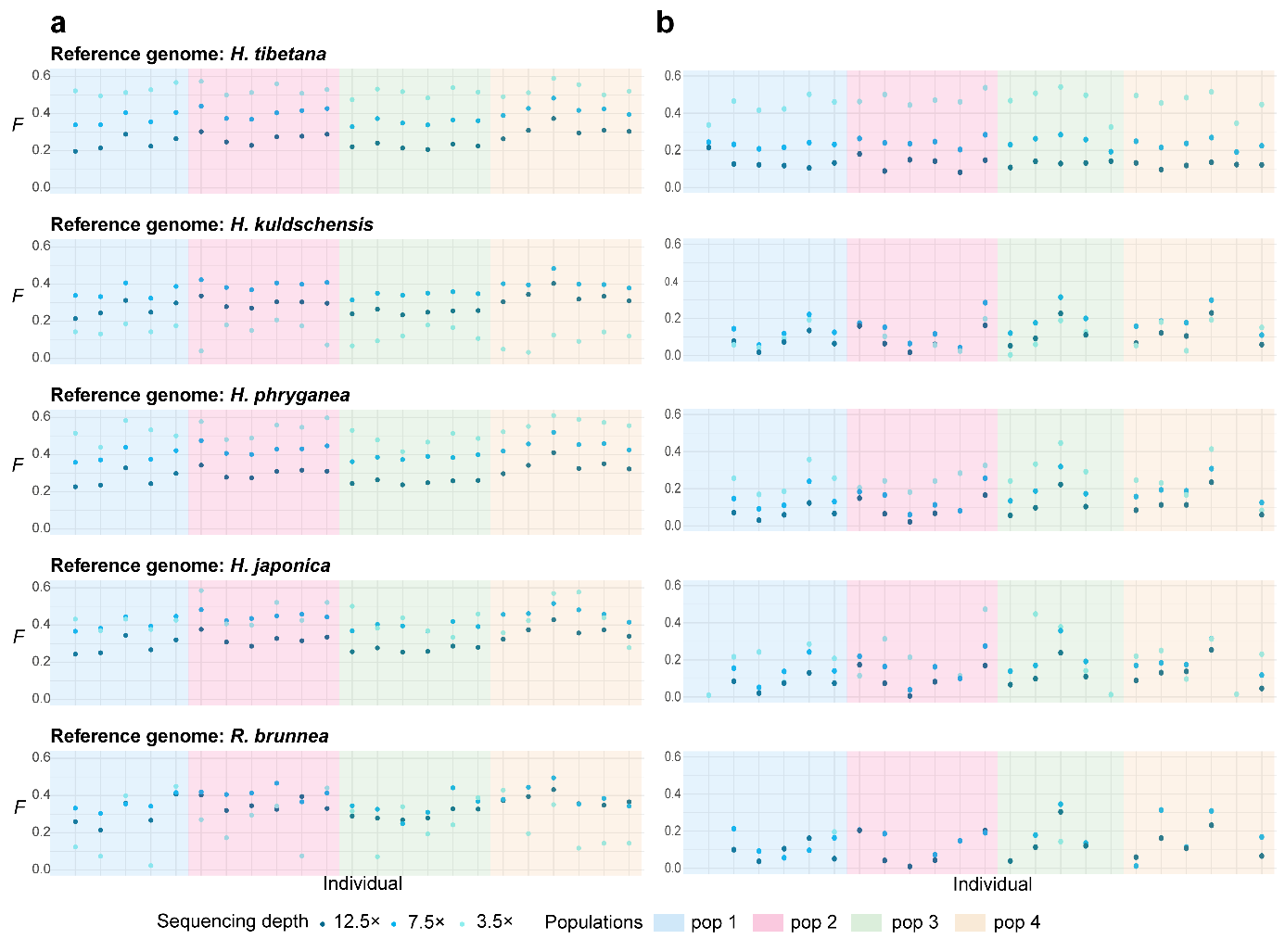


**Supplementary Figure 13: Inbreeding coefficient of each individual of (a) *H. digitata* and (b) *H. tibetana* populations depending on different RG and different sequencing depth calculated by *VCFtools*.**

The estimates of π based on the 30 datasets varied between *H. digitata* and *H. tibetana* (Supplementary Figure 15, Supplementary Figure 16). The genetic variation of all four populations were similar within each species, including the median π value and genome-wide distribution patterns. Additionally, each of the populations included a number of anomalous π values, which were represented as outliers in the plots of Supplementary Figure 15. For both *H. digitata* and *H. tibetana* populations, when mapped to a more distantly related RG or a lower sequencing depth, the π value tended to be lower, as well as the range of outliers narrowed (Supplementary Figure 15). To conclude, similar to the estimates of *F*, selecting a more closely related RG or a higher sequencing depth may improve the resolution of the results in a genetic variation estimation.


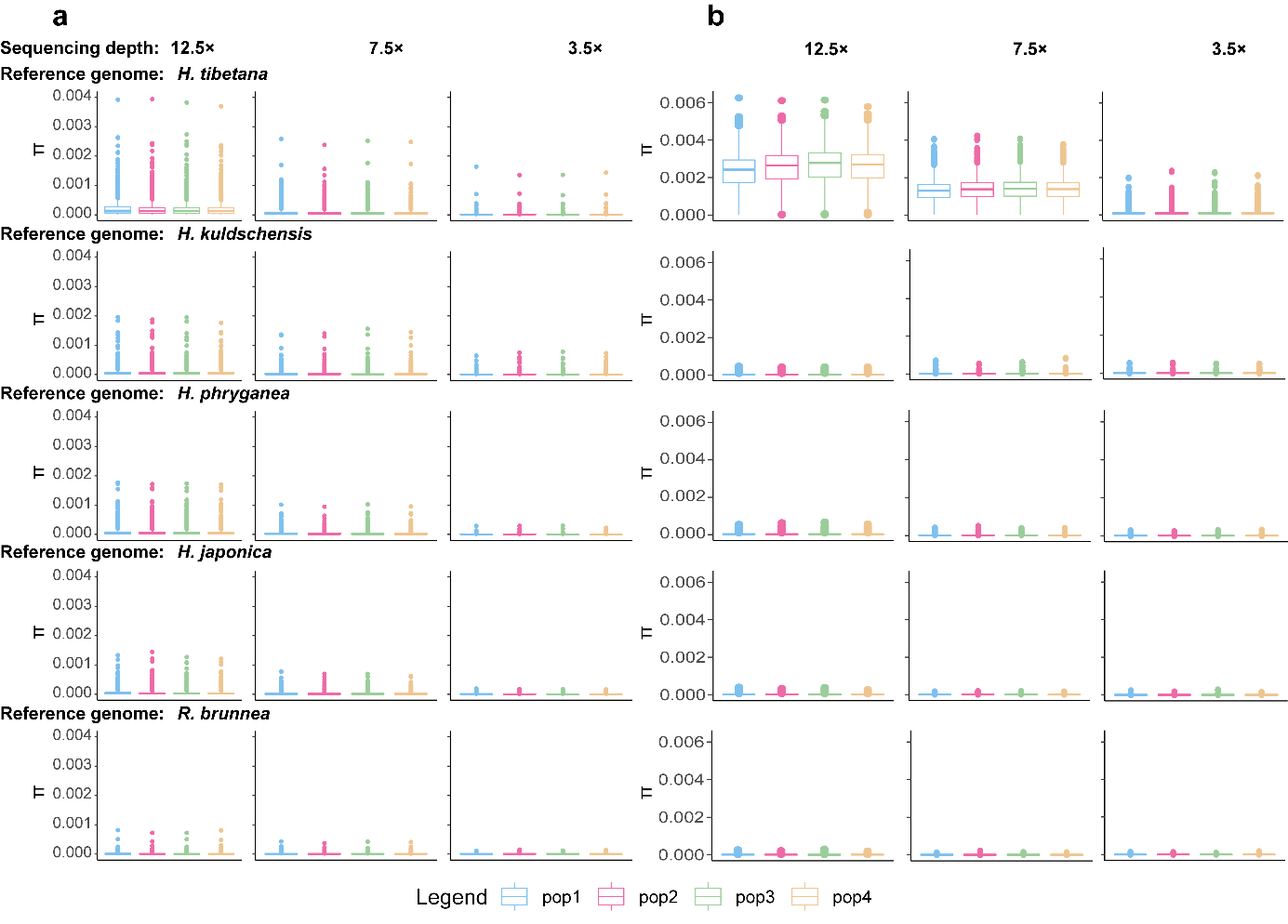


**Supplementary Figure 14:** **Nucleotide diversity of each population of (a) *H. digitata* and (b) *H. tibetana* depending on different RG and different sequencing depth calculated by *VCFtools*.**

The pairwise *F*_ST_ estimates based on most of the datasets were concordant (Supplementary Material 3). For *H. digitata*, the genetic differentiation was highest between pop 1 and pop 3 (e.g., weighted *F*_ST_ = 0.0335, RG: *H. tibetana* & depth: 12.5×), as well as between pop 1 and pop 4 (e.g., weighted *F*_ST_ = 0.0331, RG: *H. tibetana* & depth: 12.5×), while the genetic differentiation of other population pairs (pop 2 vs pop 3, pop 2 vs pop 4, pop 3 vs pop 4) was much lower (*F*_ST_ < 0.005). For *H. tibetana*, the genetic differentiation was highest between pop 1 and pop 3 (e.g., weighted *F*_ST_ = 0.17, RG: *H. tibetana* & depth: 12.5×), as well as between pop 1 and pop 4 (e.g., weighted *F*_ST_ = 0.16, RG: *H. tibetana* & depth: 12.5×), and lowest between pop3 and pop4 (e.g., weighted *F*_ST_ = ~ 0, RG: *H. tibetana* & depth: 12.5×). This pattern was observed with most of the datasets except those with low depth (3.5×) and distantly related RG (*H. phryganea* or *R. brunnea*). For example, the highest genetic differentiation among populations of *H. tibetana* was between pop 1 and pop 2 (also between pop 2 and pop 4) when using 3.5× as depth and *R. brunnea* as RG. Meanwhile, for both *H. digitata* and *H. tibetana*, the pairwise *F*_ST_ values clearly decreased when estimated with a lower sequencing depth or a further related RG. Similar to the pairwise *F*_ST_ estimates, the genome-wide *F*_ST_ estimates were affected by sequencing depth and RG for both species (3.5×, Fig. 5, Supplementary Material 3): the global *F*_ST_ values remarkably decreased with low depth (3.5×), and more discretely distributed across the whole genome. In addition, for the populations of *H. digitata*, the number of specific abnormal *F*_ST_ values across the whole genome increased either with lower depth or more distantly related RG. But for the populations of *H. tibetana*, the abnormal *F*_ST_ values decreased either with lower depth or more distantly related RG. This is likely due to the fact that the threshold value for the populations of *H. tibetana* was obtained from the dataset mapped to the conspecific RG, which might lead to a larger amount of high *F*_ST_ values, thus increasing the value of abnormal threshold. In general, for genomic pairwise *F*_ST_ estimation, based on the inherent genetic variation, selecting a more closely related RG or increasing the sequence depth range may thus improve the accuracy of the results.

We observed variable patterns of population structure in the principal component analyses among treatments (Fig. 3). The percentage of variance explained by the top three principal components among populations of *H. tibetana* was ~42%, while it was lower in *H. digitata* (~34%). The populations of both species formed distinct clusters in the PCA estimates based on the dataset mapped to *H. tibetana* with 12.5× depth. Specifically, for *H. digitata* populations, the plot using 12.5× as depth and *H. tibetana* as RG showed that pop 1 formed a distinct cluster far apart from the other three populations, pop 3 and pop 4 were mixed, and pop 2 formed an identical cluster and adjoined pop3 and pop4 (Fig. 3a). For *H. tibetana* populations, we observed more distinct clusters compared with *H. digitata* populations (Fig. 3b). The plot based on the most closely related RG and highest depth (*H. tibetana* & 12.5×) shows that pop 3 and pop 4 were clustered together, pop 1 and pop 2 formed distinct clusters respectively, and all these three clusters were separated from each other. Generally, there was a visible influence from both the sequencing depth and RG on the estimates of PCA. In particular, the resolution of the plots was noticeably decreased when selecting a more distantly related RG or a lower depth, especially for *H. digitata*. For instance, for populations of *H. digitata*, when using *H. tibetana* as RG, regardless of the depth, the cluster of pop 1 was well defined; when using *H. phryganea* as RG, pop 1 formed a distinct cluster only with 12.5× and 7.5× depth; when using *R. brunnea* as RG, no distinguishable structure was shaped even with the 12.5× depth. As for the populations of *H. tibetana*, we observed more distinct clusters compared with *H. digitata* populations (Fig. 3b). More specifically, the individuals formed distinct clusters with most of the datasets, except using *R. brunnea* as RG, which demonstrated that the effects of RG and sequencing depth on PCA analyses were different for the populations of *H. tibetana* and *H. digitata*.

We inferred the individual admixture proportions by setting K as 2, 3, and 4 (Skotte et al., 2013). For *H. digitata* populations, with the most ideal dataset (*H. tibetana* as RG and 12.5× as depth), we did not detect admixture in pop 1 across all K values; pop 2 was genetically admixed regardless of K value; all individuals in pop 3 and pop 4 were homogeneous when K = 2, but admixed when K = 3 or 4 (Fig. 6a). Hence, it indicated that pop 1 was a homogeneous population, while pop 2, pop 3, and pop 4 were represented as genetic mixtures. For *H. tibetana* populations, pop 1 and pop 2 showed the same genetic background when K was 2 but split into two different homogeneous groups when K increased; Pop 3 and pop 4 showed the same non-admixed background in most of the scenarios (Fig. 6b). Similar to the PCA estimates, the admixture analyses showed much the same trend facing the impacts of RG and depth. Take the populations of *H. digitata* as an example, when using *H. sp.* (*kuldschensis* group) as RG with decreasing the depth from 12.5× to 3.5×, pop 1 was not anymore shown as a homogeneous ancestry regardless of K value; when using 7.5× as depth and changing the RG from *H. tibetana* to *R. brunnea*, the admixture scenarios became less identical regardless of K value. Furthermore, the admixture results of *H. tibetana* were more concordant across the different RGs and depths (except with *R. brunnea* as RG), which means, that the populations of *H. tibetana* were less sensitive to the impacts of decreasing depth or using a non-conspecific RG.

**
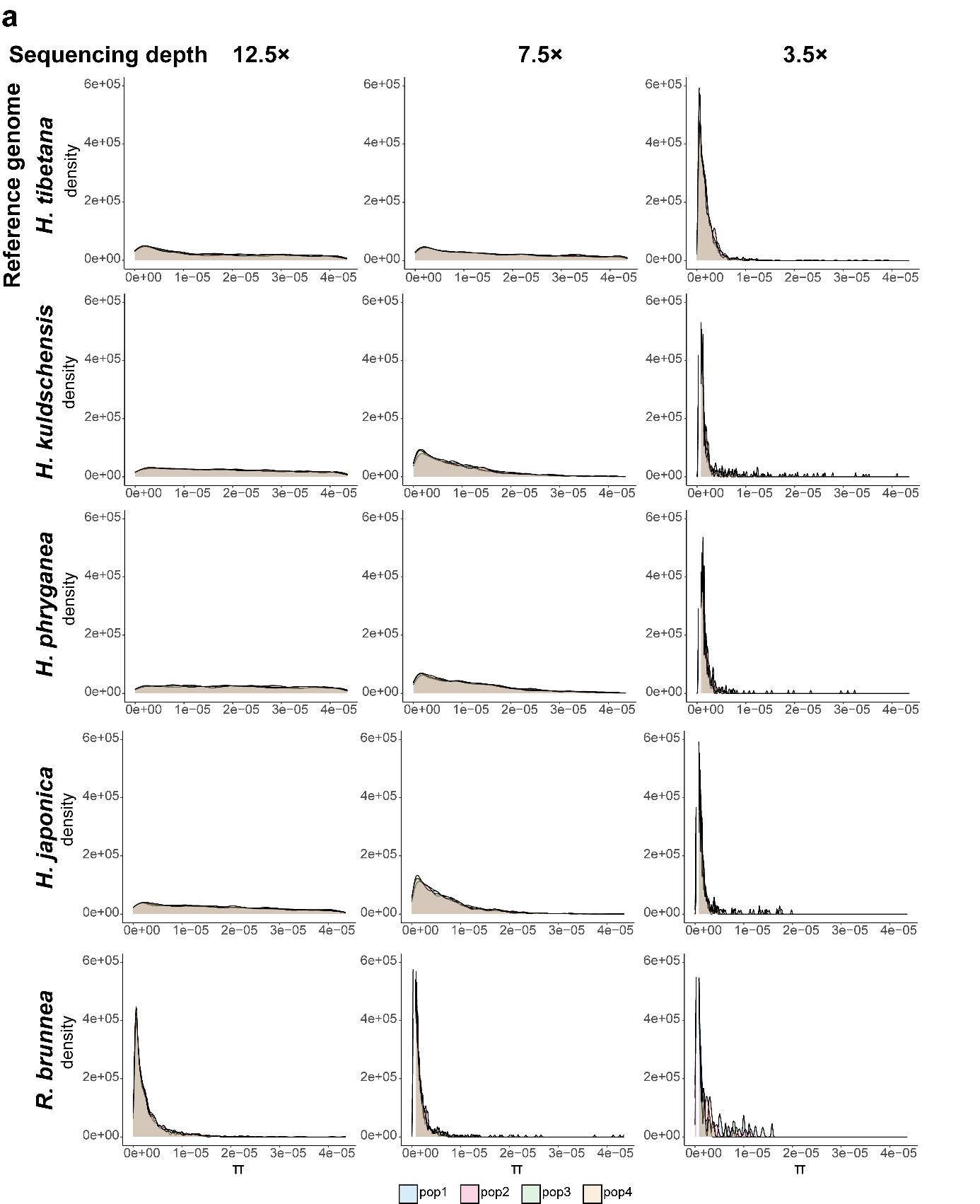
**

**
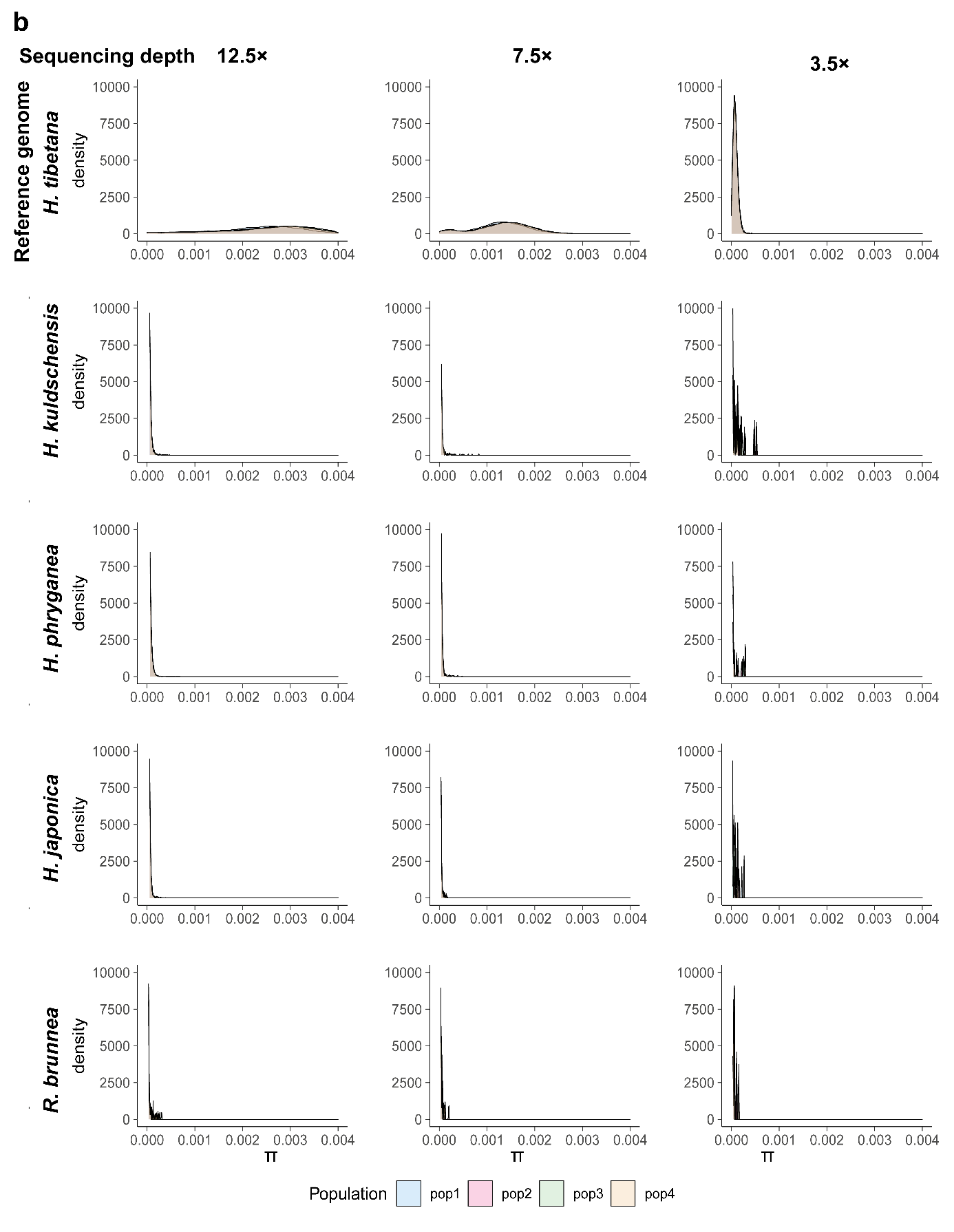
**

**Supplementary Figure 15: Density of genome-wide π value of (a) *H. digitata* and (b) *H. tibetana* populations.**

# Reference

Bolger, A. M., Lohse, M., & Usadel, B. (2014). Trimmomatic: a flexible trimmer for Illumina sequence data. *Bioinformatics, 30*(15), 2114-2120. <https://github.com/usadellab/Trimmomatic>

Danecek, P., Bonfield, J. K., Liddle, J., Marshall, J., Ohan, V., Pollard, M. O., Whitwham, A., Keane, T., McCarthy, S. A., & Davies, R. M. (2021). Twelve years of SAMtools and BCFtools. *Gigascience, 10*(2), giab008.

Fox, E. A., Wright, A. E., Fumagalli, M., & Vieira, F. G. (2019). ngsLD: evaluating linkage disequilibrium using genotype likelihoods. *Bioinformatics, 35*(19), 3855-3856. <https://github.com/fgvieira/ngsLD>

Laetsch, D. R., & Blaxter, M. L. (2017). BlobTools: Interrogation of genome assemblies. *F1000Research, 6*(1287), 1287.

Li, H. (2013). Aligning sequence reads, clone sequences and assembly contigs with BWA-MEM. *arXiv preprint arXiv:1303.3997*.

Loman, N. J., & Quinlan, A. R. (2014). Poretools: a toolkit for analyzing nanopore sequence data. *Bioinformatics, 30*(23), 3399-3401.

Marçais, G., & Kingsford, C. (2011). A fast, lock-free approach for efficient parallel counting of occurrences of k-mers. *Bioinformatics, 27*(6), 764-770.

Martin, M. (2011). Cutadapt removes adapter sequences from high-throughput sequencing reads. *EMBnet. journal, 17*(1), 10-12. <https://github.com/marcelm/cutadapt>

Ranallo-Benavidez, T. R., Jaron, K. S., & Schatz, M. C. (2020). GenomeScope 2.0 and Smudgeplot for reference-free profiling of polyploid genomes. *Nature Communications, 11*(1), 1-10. <https://github.com/tbenavi1/genomescope2.0>

Rohland, N., & Reich, D. (2012). Cost-effective, high-throughput DNA sequencing libraries for multiplexed target capture. *Genome research, 22*(5), 939-946.

Vurture, G. W., Sedlazeck, F. J., Nattestad, M., Underwood, C. J., Fang, H., Gurtowski, J., & Schatz, M. C. (2017). GenomeScope: fast reference-free genome profiling from short reads. *Bioinformatics, 33*(14), 2202-2204. <https://github.com/tbenavi1/genomescope2.0>

Waldvogel, A. M., Wieser, A., Schell, T., Patel, S., Schmidt, H., Hankeln, T., Feldmeyer, B., & Pfenninger, M. (2018). The genomic footprint of climate adaptation in Chironomus riparius. *Molecular ecology, 27*(6), 1439-1456. <https://github.com/schellt/autotrim>

Wood, D. E., Lu, J., & Langmead, B. (2019). Improved metagenomic analysis with Kraken 2. *Genome biology, 20*(1), 1-13.

Wood, D. E., & Salzberg, S. L. (2014). Kraken: ultrafast metagenomic sequence classification using exact alignments. *Genome biology, 15*(3), 1-12.
